# Wheat Small GTPase Gene *TaRABH1bL* Is Involved in High‐Temperature All‐Stage Resistance to *Puccinia striiformis* f. sp. *tritici*

**DOI:** 10.1111/mpp.70132

**Published:** 2025-08-07

**Authors:** Yifeng Shi, Xiyue Bao, Hai Li, Yuxiang Li, Xianming Chen, Xiaoping Hu

**Affiliations:** ^1^ State Key Laboratory of Crop Stress Resistance and High‐Efficiency Production, Key Laboratory of Plant Protection Resources and Pest Integrated Management of Ministry of Education, Key Laboratory of Integrated Pest Management on Crops in Northwestern Loess Plateau of Ministry of Agriculture and Rural Affairs, and College of Plant Protection Northwest A&F University Yangling China; ^2^ State Key Laboratory for Biology of Plant Diseases and Insect Pests, Institute of Plant Protection Chinese Academy of Agricultural Sciences Beijing China; ^3^ Agricultural Research Service, United States Department of Agriculture, Wheat Health, Genetics, and Quality Research Unit and Department of Plant Pathology Washington State University Pullman WA USA

**Keywords:** ERF transcription factor, high‐temperature all‐stage resistance, small GTPase, stripe rust

## Abstract

As the largest subfamily of small GTPases, the Rab subfamily plays a pivotal role in regulating biotic and abiotic stresses in plants. However, the functions of Rabs in resistance to wheat stripe rust caused by *Puccinia striiformis* f. sp. *tritici* (Pst) remain unclear. Here, we identified a Rab subfamily gene, *TaRABH1bL*, from Xiaoyan 6 (XY6), a wheat cultivar known for non‐race‐specific and durable high‐temperature all‐stage (HTAS) resistance to stripe rust. The expression level of *TaRABH1bL* was exclusively up‐regulated with Pst inoculation under the relatively high‐temperature treatment, which indicated that *TaRABH1bL* might concurrently respond to both biotic and abiotic stress signals. The *TaRABH1bL* gene was primarily expressed in leaves. Barley stripe mosaic virus (BSMV)‐induced *TaRABH1bL* gene silencing significantly reduced HTAS resistance to Pst, resulting in increased sporulation. Transient expression of *TaRABH1bL* in *Nicotiana benthamiana* leaves and wheat protoplasts confirmed its subcellular localisation in both cytoplasm and nuclei. The GTP‐binding state of TaRABH1bL (TaRABH1bL^Q69L^) exclusively interacted with the transcription factor ethylene‐responsive transcription factor 1‐like (TaERF1L) in nuclei. TaERF1L directly bound to and suppressed the activity of the GCC‐box motif, and this inhibitory effect was enhanced by the exclusive interaction between TaRABH1bL^Q69L^ and TaERF1L. Silencing *TaERF1L* significantly reduced HTAS resistance. These results suggested that under dual signals of Pst infection and relatively high temperature treatment, TaRABH1bL transferred into its GTP‐binding state and interacted with TaERF1L. Additionally, TaRABH1bL^Q69L^ enhanced the suppression of TaERF1L on its downstream susceptible or temperature‐sensitive genes containing the GCC‐box motif, thereby activating HTAS resistance to Pst in XY6.

## Graphical abstract

Under dual treatment of *Puccinia striiformis* f. sp. *tritici* (Pst) infection and relatively high temperature treatment, TaRABH1bL^Q69L^ (GTP‐binding state of TaRABH1bL) interacts with TaERF1L. TaRABH1bL^Q69L^ enhances the suppression of TaERF1L on its downstream genes containing the GCC‐box motif, thereby activating high‐temperature all‐stage (HTAS) resistance to Pst.

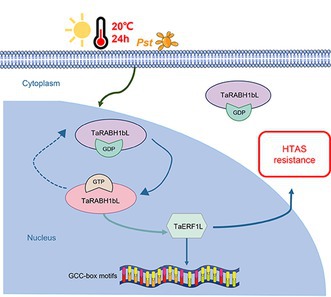

## 1 Introduction

Wheat stripe rust, an airborne fungal disease caused by *Puccinia striiformis* f. sp. *tritici* (Pst), is a major disease threatening wheat production worldwide (Hu, Hu, et al. 2022; Chen 2020; Li et al. 2023). Developing and planting resistant cultivars is the most effective and environmentally friendly method to control stripe rust. However, race‐specific resistance is likely to become ineffective due to the rapid evolution of Pst (Wang et al. 2022). Compared to many other fungal pathogens, Pst adapts to relatively cool environments. As primary hosts, wheat and its related grass species possess different types of resistance. Some types of resistance are temperature‐sensitive, and the level of resistance increases as temperature increases. Temperature‐sensitive resistance can be further categorised into high‐temperature adult‐plant (HTAP) resistance and high‐temperature all‐stage (HTAS) resistance, and the latter is also known as high‐temperature seedling‐plant (HTSP) resistance (Shang 1998; Chen 2013). Wheat cultivars with HTAP resistance are susceptible to stripe rust at the seedling stage and become resistant or more resistant when the weather becomes warm and plants grow older (Chen 2005, 2013). Under greenhouse conditions, plants at the boot to flowering stages are usually inoculated with Pst and grown at diurnal temperature cycles of 10°C–30°C to identify HTAP resistance (Chen 2013). Up to now, 11 named *Yr* genes and many quantitative trait loci (QTLs) linked to HTAP resistance have been identified and have been successfully used for controlling stripe rust in the United States (Akin et al. 2016). In contrast, HTAS resistance expresses immune responses at the seedling stage after Pst infection when plants are exposed to relatively high temperature treatments (Hu, Su, et al. 2022; Tao et al. 2018; Wang et al. 2021). Xiaoyan 6 (XY6), a wheat cultivar with HTAS resistance to stripe rust, has maintained its resistance for more than 40 years in China. The expression of HTAS resistance results in decreased uredinia on the surface of host leaves, increased necrotic host cells at infection points, and inhibition of the hyphal expansion of Pst. The optimal conditions for HTAS resistance activation are 20°C for 24 h (192 h post‐inoculation) (Tao et al. 2018; Hu, Su, et al. 2022). However, the molecular mechanisms of HTAS resistance are still not clear. An in‐depth study on the molecular mechanisms underlying HTAS resistance should be useful in guiding the development of wheat cultivars with durable resistance to stripe rust.

Plants recognise and defend against pathogens through a two‐layer defence system (Chisholm et al. 2006; Jones and Dangl 2006). The first layer is pattern‐triggered immunity (PTI), which is activated by pathogen‐associated molecular patterns (PAMPs) including bacterial flagellin, fungal xylanase and chitin (Boller and Felix 2009). Pathogens inhibit PTI and interfere with plant immunity by secreting effector proteins into plant cells. In response, plants launch the second layer of immune response called effector‐triggered immunity (ETI) to restrain the pathogen effector proteins, which is mainly based on the recognition of NBS‐LRR immune receptors (Wan et al. 2021). Despite having different activation mechanisms, PTI and ETI eventually trigger many similar downstream immune responses (Ngou et al. 2022; Yuan, Jiang, et al. 2021). An updated model of plant immune mechanisms is proposed in which these two defence systems are complementary, PTI and ETI working together to regulate plant immunity (Yuan, Ngou, et al. 2021). HTAP resistance is usually related to PTI immune responses. The wheat kinase START1 (WKS1), which is encoded by *Yr36* (a gene conferring HTAP resistance in wheat), has been shown to target and phosphorylate the thylakoid‐associated ascorbate peroxidase (tAPX) and promote typical PTI responses including cell death and reactive oxygen species (ROS) accumulation (Gou et al. 2015). In wheat cultivar XY6, HTAS resistance involves both PTI and ETI immune responses. Previous laboratory studies found that nucleotide‐binding site (NBS)‐LRR protein TaRPM1 initiates the downstream PTI immune responses by monitoring the changes in the phosphorylation level of papain‐like cysteine protease TaPLCP1 induced by receptor‐like cytoplasmic kinase TaRIPK during the occurrence of HTAS resistance in wheat; effector protein PSTG_01766 exhibits competitive interaction with TaPLCP1. Meanwhile, TaRPM1 responds to the HTAS resistance by initiating a more intense ETI response by recognising and inhibiting the expression of PSTG_01766 (Wang et al. 2019; Hu et al. 2021).

The small GTPases superfamily, function as ‘molecular switches’, cycling between the active (GTP‐binding) and inactive (GDP‐binding) states to transduce signals in eukaryotes (Wennerberg et al. 2005; Reiner and Lundquist 2018). This superfamily is categorised into Ras, Rho, Rab, Arf/Sar1 and Ran subfamilies (Tian et al. 2024). As the largest subfamily of small GTPases, the Rab subfamily plays a key role in regulating biotic and abiotic stresses in plants. Overexpression and gene silencing assays have shown that StRAB5b in the Rab subfamily positively regulates resistance to *Phytophthora infestans* in potato (Tian et al. 2023). A plant‐specific *RAB5* gene, *ARA6*, is responsible for starch and sugar content homeostasis by regulating the function of the *Qua‐Quine Starch* (*QQS*) gene in *Arabidopsis thaliana* (Tsutsui et al. 2015). In rice, *OsRab11* transgenic plants exhibit enhanced resistance to *Pseudomonas syringae* pv. *tomato* DC3000 by promoting the expression of jasmonic acid (JA)‐responsive genes and NADPH consumption by interacting with 12‐oxophytodienoic acid reductase 8 (OsOPR8) (Hong et al. 2013). In *Arabidopsis*, *RabE* transgenic plants exhibit a dwarf phenotype, characterised by smaller leaves and a reduced overall plant size. In contrast, overexpression of the *RabE1d* gene enhances plant resistance to *P. syringae* pv. *tomato* DC3000 infection (Speth et al. 2009).

The APETALA2/Ethylene responsive factor AP2/ERF is one of the largest families of transcription factors (TFs) in plants. Nakano et al. (2006) categorised the 147 AP2/ERF members of *Arabidopsis* into four subfamilies: AP2, ERF, RAV and Soloist. Members of the AP2 subfamily contain two highly similar AP2 domains, whereas members of the ERF subfamily contain only one (Chen et al. 2022). In addition, the RAV subfamily TFs contain both AP2 and B3 domains, and the Soloist subfamily TFs contain a unique AP2 domain (Feng et al. 2020). ERFs are widely involved in the regulation of abiotic stress in plants. Overexpression of the *OsERF115*/*AP2EREBP110* gene significantly upregulates the expression level of *P5CS1*, a proline biosynthesis gene, to enhance drought tolerance in rice (Park et al. 2021). ROS serve as signal molecules that initiate stress responses and facilitate signal transduction, while ERFs enhance plant resilience under stressful conditions by improving the capacity for ROS clearance (Hong et al. 2020). In *Nicotiana tabacum* (tobacco), NtERF172 binds to the promoter region of the catalase gene *NtCAT* to activate its expression. Exogenous application of a catalase inhibitor on the *NtERF172*‐overexpression plants results in increased levels of H_2_O_2_, indicating that *NtERF172* positively regulates endogenous catalase‐mediated H_2_O_2_ content homeostasis (Zhao et al. 2020). ERFs also play a crucial role in regulating biotic stress in plants. By interacting with MdERF114, MdMYB8 enhances the binding ability of MdERF114 to the *MdPRX63* promoter, thereby increasing lignin deposition and enhancing resistance to *Fusarium solani* in apple (Liu et al. 2023). In *Arabidopsis*, ERF114 positively modulates plant defence responses including accumulation of lignin and mediates PevD1 (a *Verticillium dahliae* secretory effector)‐induced disease resistance to *P. syringae* pv. *tomato* DC3000 (Li, Liu, et al. 2022; Li, Zhang, et al. 2022).

A large number of research studies have demonstrated that small GTPases play a critical role in regulating biotic and abiotic stresses, which is consistent with the dual signals of high temperature treatment (abiotic stress) and Pst infection (biotic stress) for activating HTAS resistance in XY6. However, the potential role of small GTPases in activating HTAS resistance remains unclear. In this study, we identified a small GTPase gene, *TaRABH1bL*, through transcriptomics sequencing of Pst‐infected XY6 seedlings under different temperature treatments (Tao et al. 2018). The expression level of *TaRABH1bL* was significantly up‐regulated under simultaneous biotic and abiotic stress conditions. A barley stripe mosaic virus (BSMV)‐induced gene silencing assay demonstrated that *TaRABH1bL* positively regulated HTAS resistance. The GTP‐binding state of TaRABH1bL interacted with the biotic and abiotic stresses regulator TaERF1L in nuclei. This interaction enhanced the inhibitory effect of TaERF1L on the transcriptional activity of genes containing a GCC‐box motif. Silencing *TaERF1L* compromised HTAS resistance. Our results suggest that under the biotic (Pst infection) and abiotic (the relatively high temperature treatment) stress conditions, the GTP‐binding state of TaRABH1bL interacted with TaERF1L in nuclei. Additionally, this interaction enhanced the suppression of TaERF1L on its downstream susceptible or temperature‐sensitive genes containing the GCC‐box motif, thereby activating the HTAS resistance to stripe rust in XY6.

## 2 Results

### 2.1 Identification and Sequence Analysis of *TaRABH1bL*

Small GTPases act as key coordinators in enabling plants to cope with biotic and abiotic stresses. In wheat cultivar XY6, the activation of HTAS resistance requires both biotic (Pst infection) and abiotic (relatively high temperature treatment) signals. However, the potential involvement of small GTPases in HTAS activation remains elusive. In our previous transcriptomics analysis of XY6 inoculated with Pst under two different temperature treatments, three small GTPase genes were identified (Figure S1). Notably, a Rab subfamily gene *TaRABH1bL* was highly up‐regulated under the normal‐high‐normal temperature (NHN, the XY6 were kept at constant 15°C until 192 h after Pst inoculation, transferred to 20°C for 24 h, then back to 15°C to activate HTAS resistance) treatment compared to the normal temperature (N, the XY6 were kept at constant 15°C after Pst inoculation) treatment. Therefore, we assumed that *TaRABH1bL* functions as the key regulator for linking temperature and pathogen signalling in HTAS resistance. Subsequently, a gene fragment containing the 645‐bp open reading frame (ORF) was cloned from XY6. The gene shares 100% amino acid identity with the RABH1b‐like protein in wheat. TaRABH1bL protein was predicted to contain a single RAB domain based on SMART (http://smart.embl‐heidelberg.de) analysis (Figure S2a). Further BLAST search on the wheat UGRI genome database showed that *TaRABH1bL* shares 100% nucleotide homology with *TaRABH1bL‐4B* (Figure S2b).

### 2.2 Expression Profiles of *TaRABH1bL* in Different Tissues of XY6 and in Response to Pst Infection and Temperature Treatments

**FIGURE 1:**
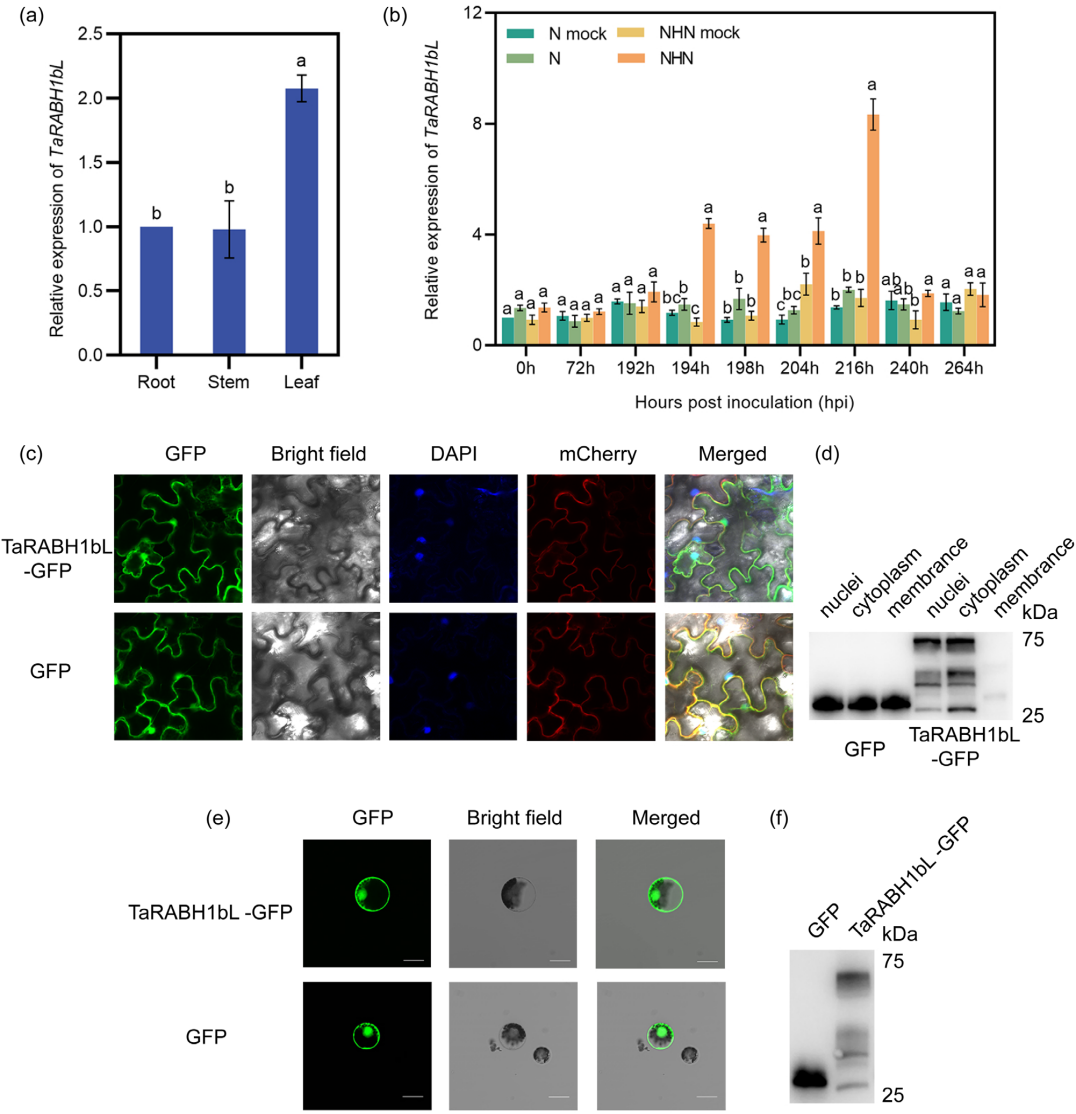
The expression levels of *TaRABH1bL* in different wheat tissues and in response to high‐temperature all‐stage (HTAS) resistance to *Puccinia striiformis* f. sp. *tritici* (Pst) in wheat cultivar Xiaoyan 6, and subcellular localisation of TaRABH1bL. (a) The transcription levels of *TaRABH1bL* in wheat roots, stems and leaves. (b) The expression level of *TaRABH1bL* upon dual induction of Pst inoculation and the relatively high temperature treatment. N mock: normal temperature (15°C) without Pst inoculation; N: normal temperature (15°C) with Pst inoculation; NHN mock: high‐temperature (20°C) for 24 h exposure at 192 h post‐inoculation (hpi) and then transferred to 15°C without Pst inoculation; NHN: high‐temperature (20°C) for 24 h at 192 hpi and then transferred to 15°C with Pst inoculation. Duncan's multiple range test (*p* < 0.05) was conducted to test the statistical significance. Three independent biological replicates were conducted for the reverse transcription‐quantitative PCR analyses. (c) Transient expression of TaRABH1bL‐GFP and green fluorescence protein (GFP) proteins in *Nicotiana benthamiana* leaves. 4′,6‐diamidino‐2‐phenylindole (DAPI) staining was used to indicate the nuclei and TaWPI6‐mCherry was used as a plasma membrane marker. Bars = 50 μm. (d) *N. benthamiana*, membranal, cytoplasmic and nuclear protein fractions were probed for the presence of TaRABH1bL‐GFP and GFP proteins by western blot using anti‐GFP antibody. (e) Transient expression of TaRABH1bL‐GFP and GFP in wheat protoplasts. Bars = 20 μm. (f) Total protein of TaRABH1bL‐GFP and GFP in wheat protoplasts were examined by western blot using anti‐GFP antibody.

Based on the reverse transcription‐quantitative PCR (RT‐qPCR) analysis, *TaRABH1bL* was primarily expressed in leaves, with expression levels 2.1 times higher than that in roots and stems (Figure 1a). To analyse the *TaRABH1bL* expression profile in HTAS resistance to Pst, the expression level of *TaRABH1bL* was estimated under two different temperature treatments (N and NHN). As shown in Figure 1b, after transferring seedlings to 20°C for 24 h at 192 h post‐inoculation (hpi), the expression level of *TaRABH1bL* under the NHN treatment gradually increased at 204 hpi, reaching a peak at 216 hpi, which was about 8.3 times higher than that at 0 hpi under the N treatment, whereas there was no significant difference in the expression level of *TaRABH1bL* between the NHN and N treatments in non‐infected leaves after the relatively high temperature treatment. In contrast, the expression level of *TaRABH1bL* showed no significant difference under the NHN and the N treatment in susceptible cultivar MX169 (Figure S3). In conclusion, the expression of *TaRABH1bL* was induced by dual signals of biotic (Pst infection) and abiotic (the relatively high temperature treatment) stresses in wheat cultivar XY6.

### 2.3 Subcellular Localisation of TaRABH1bL

In order to determine the subcellular localisation of TaRABH1bL, fusion proteins TaRABH1bL‐GFP and GFP were expressed in *Nicotiana benthamiana* leaves and wheat protoplasts. As shown in Figure 1c, the nuclei were stained blue by 4ʹ,6‐diamidino‐2‐phenylindole (DAPI) and the red fluorescence indicated the TaWPI6‐mCherry membrane marker; the green fluorescence of TaRABH1bL‐GFP did not overlap into the yellow fluorescence on the membrane. In contrast, the nuclei of cells containing GFP stained blue by DAPI, and overlapping yellow fluorescence was observed on the membrane. Nuclear, cytoplasmic and membranal proteins were examined by western blot using anti‐GFP antibody (Figure 1d). In wheat protoplasts, the green fluorescence was observed in both cytoplasm and nuclear fractions (Figure 1e). Total proteins from wheat protoplasts were extracted and analysed by western blot to confirm the successful expression of GFP and TaRABH1bL‐GFP proteins (Figure 1f). These results indicated that TaRABH1bL was located in both nuclei and cytoplasm instead of the membrane.

### 2.4 Silencing *TaRABH1bL* Significantly Reduced HTAS Resistance to Pst in XY6

**FIGURE 2:**
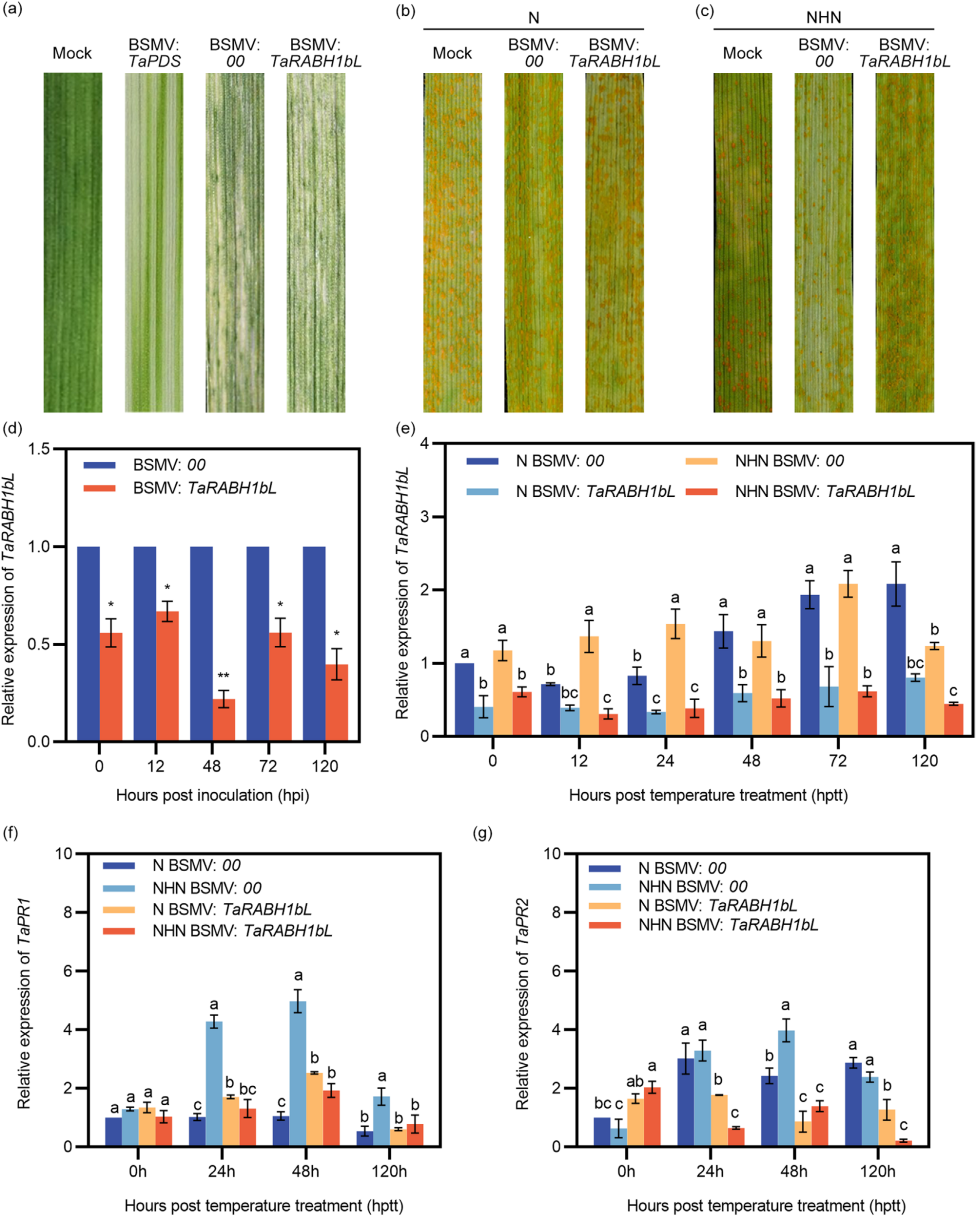
Barley stripe mosaic virus (BSMV)‐mediated gene silencing of *TaRABH1bL*. (a) Phenotypes of wheat cultivar Xiaoyan 6 (XY6) leaves 10 days after BSMV inoculation. Mock, wheat seedlings inoculated with the 1× FES buffer; phenotypes of XY6 leaves inoculated with Pst 14 days after inoculation under the N treatment (b) and the NHN treatment (c); (d) silencing efficiency of *TaRABH1bL* in BSMV‐infected leaves at 0, 12, 48, 72, 120 h post‐inoculation (hpi). Single asterisk indicates significant differences at the *p* < 0.05 level while double asterisks indicate significant differences at the *p* < 0.01 level in the Student's *t* test; (e) The expression levels of *TaRABH1bL* in the BSMV:00 or BSMV:*TaRABH1bL* leaves under the N and NHN treatments at 0, 12, 24, 48, 72 and 120 h post‐temperature treatment (hptt). The expression levels of *TaPR1* (f) and *TaPR2* (g) at 0, 24, 48 and 120 hptt. Duncan's multiple range test (*p* < 0.05) was conducted to determine significant differences. Three independent biological replicates were conducted for the reverse transcription‐quantitative PCR analyses.

**FIGURE 3:**
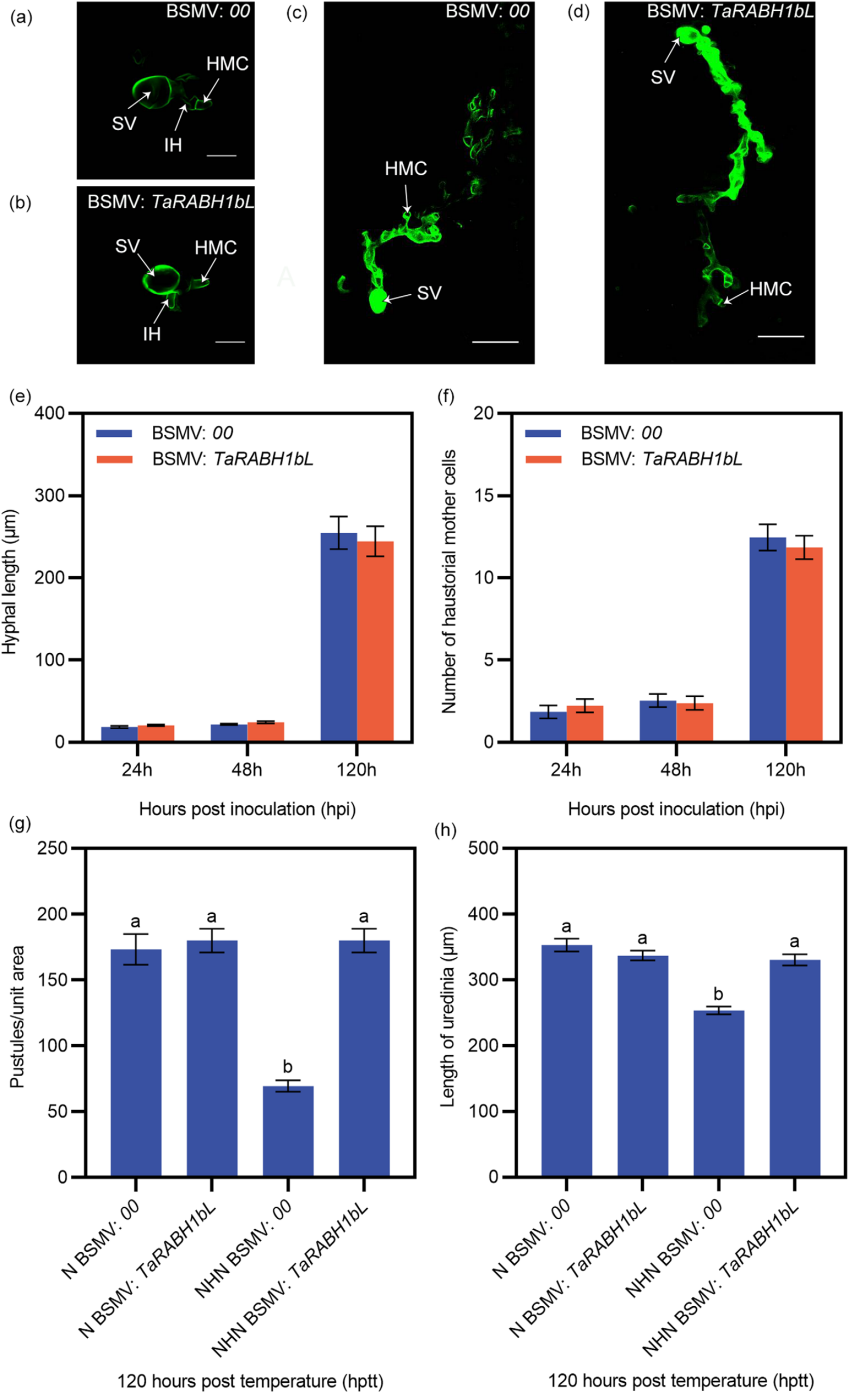
Histological observations of *TaRABH1bL*‐silenced and non‐silenced leaves inoculated with *Puccinia striiformis* f. sp. *tritici*. Hyphal developments in non‐silenced plants (a, c) and *TaRABH1bL*‐silenced plants (b, d) at 48 h post‐inoculation (hpi) (bars = 20 μm) and 120 hpi (bars = 50 μm), respectively. HMC, haustorial mother cell; IH, infection hypha; SV, substomatal vesicle. Hyphal length (e) and the number of haustorial mother cells (f) were assessed at 24, 48 and 120 hpi. The number of pustules per unit leaf area (g) and the length of uredinia (h) in the *TaRABH1bL*‐silenced and non‐silenced wheat plants under the N and NHN treatments at 120 h post‐temperature treatment (hptt). Different letters above bars indicate significant difference (Student's *t* test, *p* < 0.05).

To confirm the function of *TaRABH1bL* in HTAS resistance, we performed a barley stripe mosaic virus (BSMV)‐mediated virus‐induced gene silencing (VIGS) assay. The positive control exhibited photobleaching symptoms on wheat leaves at 10 days post‐inoculation (dpi) with BSMV: *TaPDS*, while wheat leaves inoculated with BSMV:00 and BSMV:*TaRABH1bL* showed chlorotic mosaic symptoms (Figure 2a). The expression level of the *TaRABH1bL* gene was decreased by 33.10%–78.04% in *TaRABH1bL*‐silenced plants compared to BSMV:00 plants, confirming the success of the silencing (Figure 2d). At 14 dpi with Pst race CYR32 on the fourth leaves of wheat seedlings, N‐treated wheat leaves showed susceptible phenotypes (infection types [ITs] 7–9) with dense uredinia on both BSMV:00 and BSMV:*TaRABH1bL* leaves (Figure 2b). Under the NHN treatment, BSMV:00‐inoculated wheat leaves exhibited resistant phenotypes (ITs 3–5) with limited sporulation, whereas the *TaRABH1bL*‐silenced leaves showed large amounts of uredinia and less necrosis (ITs 6–8) (Figure 2c). However, the expression level in the *TaRABH1bL*‐silenced plants remained lower than that in the non‐silenced plants under the NHN treatment (Figure 2e). The expression level of resistance genes *TaPR1* (Figure 2f) and *TaPR2* (Figure 2g) were both significantly decreased (*p* < 0.05) in *TaRABH1bL*‐silenced leaves compared with BSMV:00‐inoculated leaves under the NHN treatment. Histological observations were performed to further assess the function of *TaRABH1bL* in HTAS resistance. Statistical analyses of hyphal length and the number of haustorial mother cells showed no significant differences between BSMV:00‐ and BSMV:*TaRABH1bL‐*inoculated plants at 24, 48 and 120 hpi (Figure 3a–f). At 120 h after the high‐temperature treatment (312 hpi, also designated as 120 hptt), the number of pustules per unit leaf area and the length of uredinia under the NHN treatment was significantly lower (*p* < 0.05) than those under the N treatment in BSMV:00 ‐inoculated plants, while no significant difference was observed between the NHN and N treatments in BSMV:*TaRABH1bL*‐inoculated plants (Figure 3g,h). These results demonstrated that *TaRABH1bL* positively regulated HTAS resistance to Pst in XY6.

### 2.5 TaRABH1bL Interacted With TaERF1L

**FIGURE 4:**
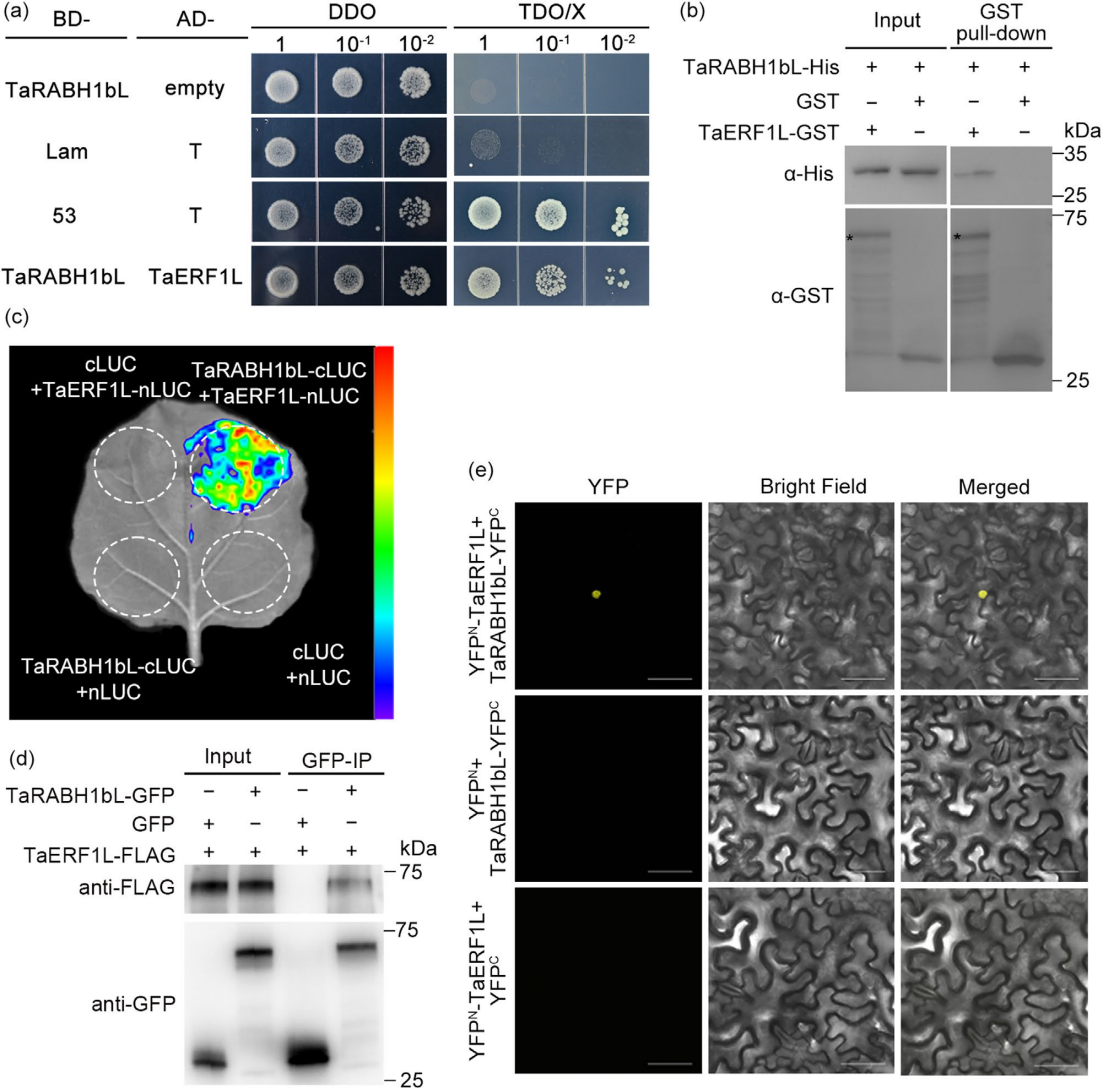
TaRABH1bL interacted with TaERF1L in vitro and in vivo. (a) TaRABH1bL interacted with TaERF1L in a yeast two‐hybrid (Y2H) assay. pGBKT7 (BD)‐53 + pGADT7 (AD)‐T and BD‐Lam + AD‐T were used as positive and negative control, respectively. (b) TaRABH1bL and TaERF1L interaction verified by pull‐down assay. TaRABH1bL‐His and TaERF1L‐GST proteins were expressed in *Escherichia coli* BL21 and incubated with GST resin. Anti‐His or anti‐GST antibody was used for western blot assay. (c) Confirmation of interaction between TaRABH1bL and TaERF1L using a split‐luciferase (LUC) complementation assay. (d) Co‐immunoprecipitation (co‐IP) assay of TaRABH1bL and TaERF1L in *Nicotiana benthamiana*. Total proteins were immunoprecipitated using GFP‐Trap beads followed by immunoblotting with anti‐FLAG and anti‐GFP antibodies. (e) Bimolecular fluorescence complementation (BiFC) assay was used to confirm the interaction between TaRABH1bL and TaERF1L in nuclei. The YFP^N^ + TaRABH1bL‐YFP^C^ and YFP^C^ + TaERF1L‐YFP^N^ construct pairs were used as negative controls. The YFP fluorescence signal was observed under a FV3000 confocal laser scanning microscope. Bars = 50 μm. Three biological replicates were performed for each protein interaction assays independently.

To further clarify the function of *TaRABH1bL* in HTAS resistance, we identified candidate interactors of TaRABH1bL by screening a yeast two‐hybrid (Y2H) cDNA library generated from Pst‐infected and the relatively high temperature‐treated leaves of XY6. After excluding the autoactivation and toxicity of binding domain (BD)‐TaRABH1bL (Figure S4), we identified a wheat ethylene‐responsive transcription factor 1‐like protein, TaERF1L, as the potential interactor of TaRABH1bL. Yeast cells carrying BD‐TaRABH1bL and AD‐TaERF1L plasmids grew normally and turned blue on SD/−Trp/−Leu/−His/+5‐bromo‐4‐chloro‐3‐indoxyl‐α‐D‐galactopyranoside (X‐α‐gal) (triple dropout, TDO/X) medium, which was consistent with the positive control (Figure 4a), suggesting an interaction between TaRABH1bL and TaERF1L in vivo. A follow‐up pull‐down assay confirmed the in vitro interaction between TaRABH1bL‐His and TaERF1L‐GST, but not between TaRABH1bL‐His and GST proteins (Figure 4b). In a split‐luciferase (LUC) complementation assay, the luminescence signal was detected in *N. benthamiana* leaves co‐expressing TaRABH1bL‐cLUC and TaERF1L‐nLUC instead of negative controls (Figure 4c). Further confirmation was performed with a co‐immunoprecipitation (co‐IP) assay. TaERF1L‐FLAG was identified in protein samples immunoprecipitated by TaRABH1bL‐GFP, but was not found in GFP‐immunoprecipitated components (Figure 4d), indicating that TaRABH1bL‐GFP interacted with TaERF1L‐FLAG in planta. Finally, we performed a bimolecular fluorescence complementation (BiFC) assay. Under the confocal microscopic observation, a yellow fluorescence signal was detected in *N*. *benthamiana* cell nuclei only when TaRABH1bL‐YFP^C^ and TaERF1L‐YFP^N^ were co‐expressed (Figure 4e).

### 2.6 Effects of Different Active States of TaRABH1bL on Its Function

Through amino acid sequence alignment of TaRABH1bL and its homologues in the NCBI database, we found that the TaRABH1bL protein contained the unique G1–G5 motifs of Rab proteins (Figure S5). Among them, mutating glutamine (Q) to leucine (L) in the G3 motif results in the active state of Rab proteins, while the mutation of asparagine (N) to isoleucine (I) in the G4 motif makes them inactive (Pinheiro et al. 2009). We generated the active (GTP‐binding) TaRABH1bL^Q69L^ and the inactive (GDP‐binding) TaRABH1bL^N122I^ to investigate the effects of different active states of TaRABH1bL on its function.

**FIGURE 5:**
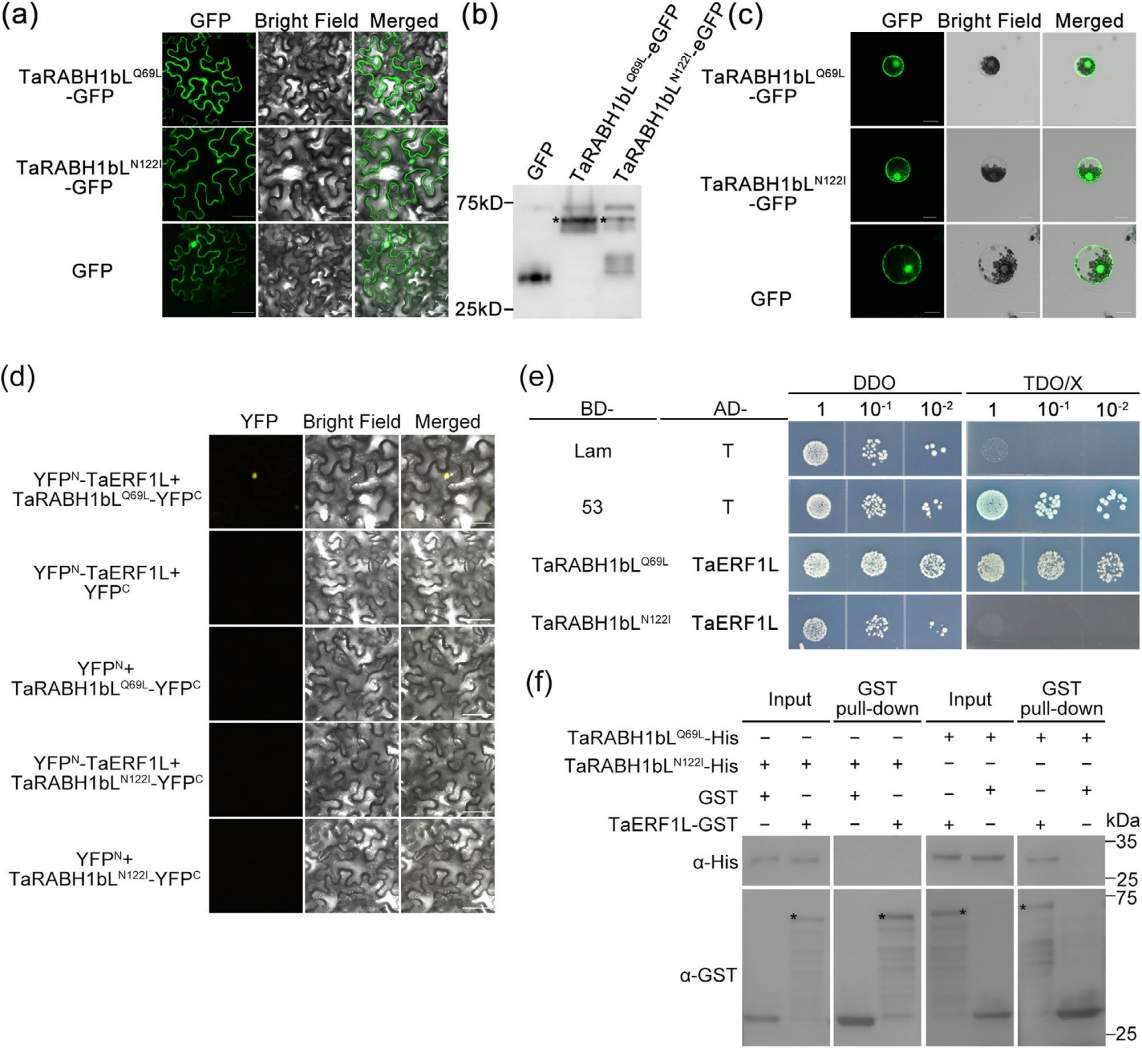
The impact of various active states of TaRABH1bL on its biological functions. (a) Transient expression of TaRABH1bL^Q69L^ (active‐state, also called the GTP‐binding state of TaRABH1bL)‐GFP, TaRABH1bL^N122I^ (inactive‐state, also called the GDP‐binding state of TaRABH1bL)‐GFP and GFP proteins in *Nicotiana benthamiana* leaves. Bars = 50 μm. (b) Western blot analysis of TaRABH1bL^Q69L/N122I^‐GFP and GFP proteins in *N. benthamiana* leaves using anti‐GFP antibody. (c) Subcellular localisation of TaRABH1bL^Q69L/N122I^ in wheat protoplasts. Bars = 20 μm. (d) Bimolecular fluorescence complementation BiFC assay examined the interactions between TaRABH1bL^Q69L/N122I^ and TaERF1L. Bars = 50 μm. (e) The interaction between TaRABH1bL^Q69L/N122I^ and TaERF1L was verified via yeast two‐hybrid (Y2H) assay. (f) Pull‐down assay examined the in vitro interactions between TaRABH1bL^Q69L/N122I^ and TaERF1L. Three biological replicates were performed for BiFC, Y2H and pull‐down assays independently.

We first observed the subcellular localisation of TaRABH1bL^Q69L^ and TaRABH1bL^N122I^ in *N. benthamiana* leaves and wheat protoplasts. The green fluorescence signal was observed in cytoplasm and nuclei, consistent with the subcellular localisation of TaRABH1bL (Figure 5a–c). We then co‐expressed TaERF1L‐RFP with GFP, TaRABH1bL‐GFP, TaRABH1bL^Q69L^‐GFP and TaRABH1bL^N122I^‐GFP in *N. benthamiana* leaves. The yellow overlapping fluorescence signal was observed only in nuclei (Figure S6). To further explore the influence of various states on the function of TaRABH1bL protein, we examined the interaction between TaRABH1bL^Q69L/N122I^ and TaERF1L protein. The BiFC assay showed that a yellow fluorescent signal in nuclei was observed only when TaERF1L‐YFP^N^ and TaRABH1bL^Q69L^‐YFP^C^ were co‐expressed in the leaves (Figure 5d). Yeast strains carrying BD‐TaRABH1bL^Q69L^ and AD‐TaERF1L plasmids grew normally and appeared blue on TDO/X medium, consistent with the positive control. However, yeast cells containing BD‐TaRABH1bL^N122I^ and AD‐TaERF1L plasmids did not grow on TDO/X medium (Figure 5e). The pull‐down assay showed similar results, confirming the in vitro interaction between TaRABH1bL^Q69L^ and TaERF1L (Figure 5f), indicating that TaERF1L interacts only with the GTP‐binding form of TaRABH1bL.

**FIGURE 6:**
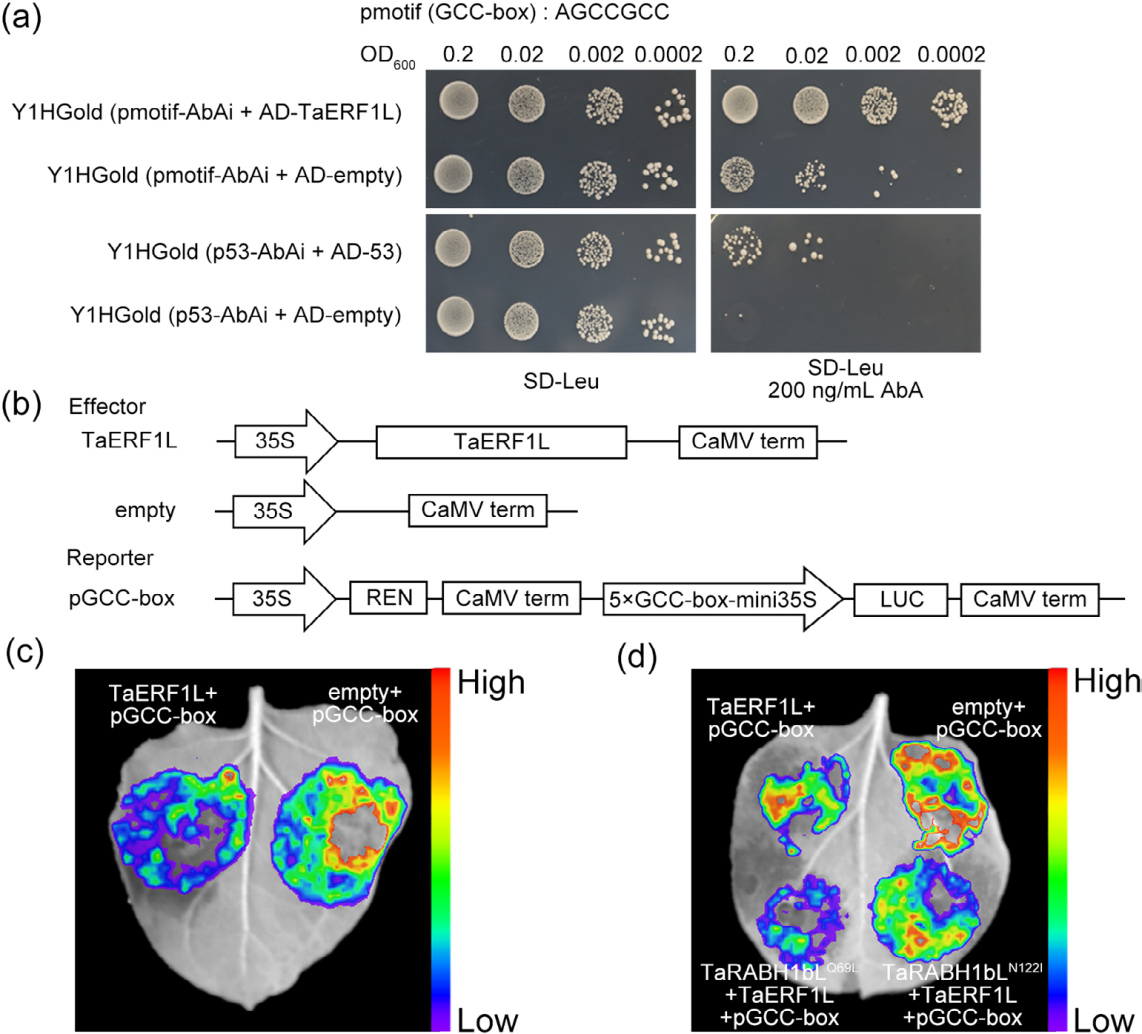
TaERF1L directly bound to and suppressed the activity of the GCC‐box motif, and this inhibitory effect was enhanced by TaRABH1bL^Q69L^ (a) Yeast one‐hybrid (Y1H) assays revealed TaERF1L bound to the GCC‐box motif. (b) A schematic diagram of the constructs used in the dual‐luciferase (LUC) assay. (c) Suppression the transcriptional activity of the GCC‐box motif by TaERF1L via dual‐LUC assay. (d) The effects of different TaRABH1bL states on TaERF1L's suppression of LUC activity in the GCC‐box motif. Three biological replicates were performed for Y1H and dual‐LUC assays independently.

Previous studies have demonstrated that AP2/ERF transcription factors (TFs) bind to the GCC‐box *cis*‐elements to regulate plant immunity (Coll et al. 2024). Yeast one‐hybrid (Y1H) assays revealed that yeast cells containing the GCC‐box motif and AD‐TaERF1L (pmotif1‐AbAi + AD‐TaERF1L) construct pairs grew on SD−Leu medium supplemented with 200 ng/mL aureobasidin A (AbA), confirming that TaERF1L could bind to the GCC‐box motif (Figure 6a). We further investigated whether TaERF1L could affect the luciferase gene expression driven by a 5 × GCC‐box‐mini35S reporter construct. In *N. benthamiana* leaves, transient expression of *TaERF1L* in combination with the firefly *LUC* gene under the control of the GCC‐box motif resulted in a significantly decreased luciferase activity (Figure 6b,c). Importantly, co‐expression of *TaRABH1bL* ^*Q69L*^ with *TaERF1L* and the GCC‐box motif led to a significantly lower luciferase activity compared to co‐expression of *TaRABH1bL* ^*N122I*^ with *TaERF1L* and the GCC‐box motif (Figure 6d). These results indicated that the inhibitory process of the TF TaERF1L on its downstream GCC‐box motif was enhanced by the interaction between TaERF1L and TaRABH1bL^Q69L^. Overall, the different active states of TaRABH1bL primarily affected its interaction with TaERF1L, rather than its subcellular localisation, with only TaRABH1bL^Q69L^ interacting with TaERF1L. Furthermore, the inhibitory effect of TaERF1L on its downstream target genes with the GCC‐box motif in their promoters was strengthened by the exclusive interaction between TaRABH1bL^Q69L^ and TaERF1L.

### 2.7 Silencing *TaERF1L* Compromised Wheat HTAS Resistance to Pst

**FIGURE 7:**
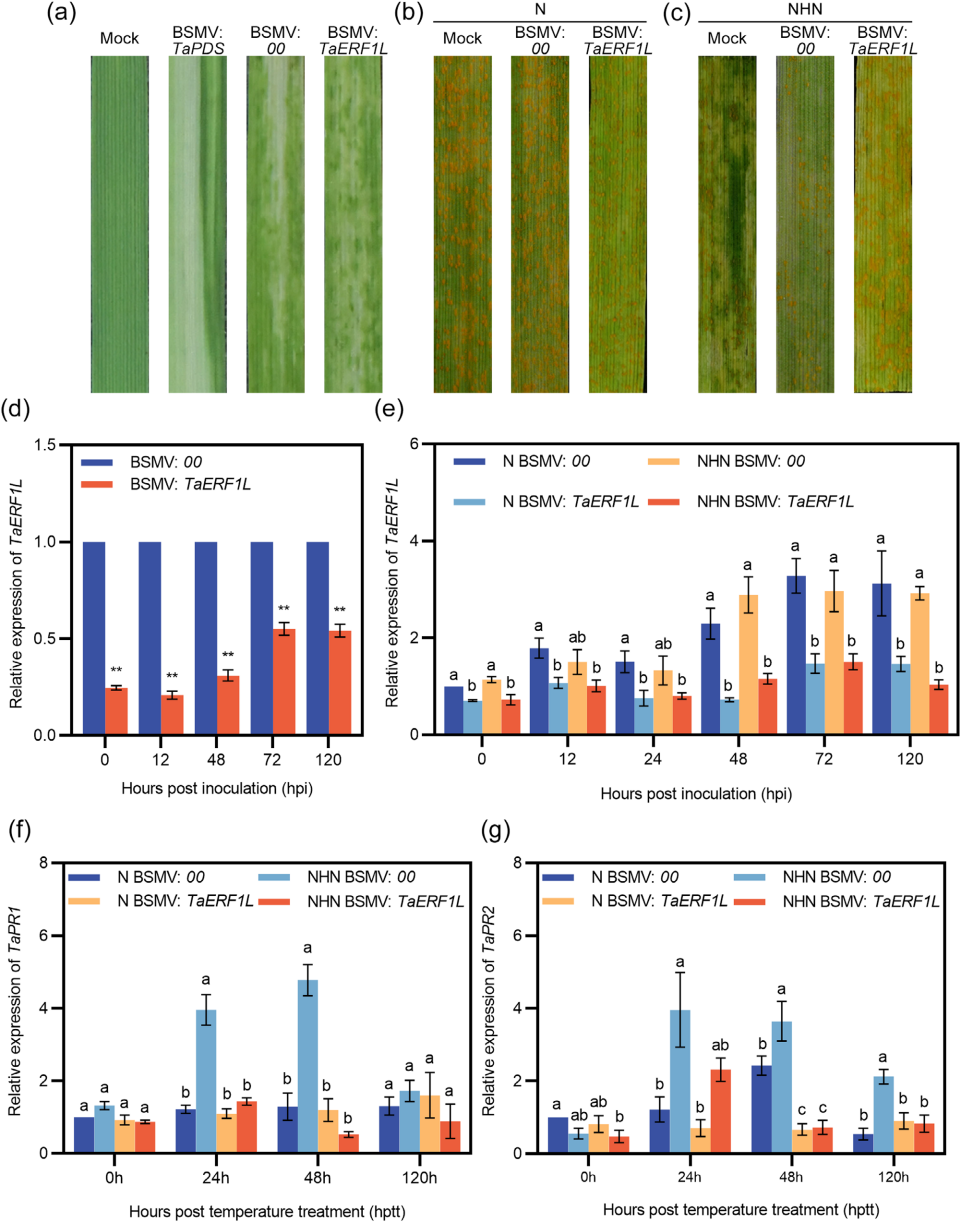
Functional analyses of *TaERF1L* in high‐temperature all‐stage (HTAS) resistance to *Puccinia striiformis* f. sp. *tritici* (Pst) using the barley stripe mosaic virus (BSMV)‐induced gene silencing assay. (a) Chlorotic mosaic symptoms and photobleaching of wheat leaves at 10 days after BSMV inoculation. Mock, wheat seedlings inoculated with the 1 × FES buffer. Phenotypes of the wheat seedlings inoculated with *Pst* at 312 h post‐inoculation (hpi) under the N treatment (b) and the NHN treatment (c); (d) Silencing efficiency assessment of *TaERF1L* at 0, 24, 48, 120 hpi. **Significant difference at *p* < 0.01 according to Student's *t* test; (e) The expression level of *TaERF1L* in the BSMV:00 or BSMV:*TaERF1L* leaves under the N and NHN treatments at 0, 12, 24, 48, 72 and 120 h post‐temperature treatment (hptt). The expression levels of *TaPR1* (f) and *TaPR2* (g) were assessed at 0, 24, 48 and 120 hptt. Different letters above bars indicate significant difference (Duncan's multiple range test, *p* < 0.05) . Three independent biological replicates were conducted for the reverse transcription‐quantitative PCR analyses.

We performed a BSMV‐mediated *TaERF1L* gene silencing assay to investigate whether TaERF1L participated in HTAS resistance to Pst in XY6. Wheat leaves inoculated with BSMV:*TaPDS* showed photobleaching at 10 dpi, while leaves inoculated with other BSMV constructs showed chlorosis symptoms (Figure 7a). Compared with BSMV:00 plants, the expression of *TaERF1L* was significantly decreased under the N and NHN treatments (Figure 7d,e), indicating that the *TaERF1L* silencing system was successfully constructed. Regarding the phenotypes, BSMV:00*‐* and BSMV:*TaERF1L‐*inoculated seedlings under the N treatment showed higher susceptibility (ITs 7–9) (Figure 7b). Under the NHN treatment, wheat seedlings inoculated with BSMV:00 showed the resistance phenotype (ITs 3–5) while BSMV:*TaERF1L*‐inoculated plants showed susceptibility with massive sporulation (ITs 6–8) (Figure 7c). The expression level of *TaPR1* (Figure 7f) and *TaPR2* (Figure 7g) was significantly reduced (*p* < 0.05) in BSMV:*TaERF1L*‐inoculated plants compared with non‐silenced plants under the NHN treatment.

Histological observations were performed to evaluate the hyphal length and the number of haustorial mother cells of Pst at 24, 48 and 120 hpi. Compared with BSMV:00*‐*inoculated plants, the *TaERF1L*‐silenced plants showed increased hyphal length and number of haustorial mother cells at 120 hpi (*p* < 0.05) (Figure S7a,b). There was no significant difference in the linear length of uredinia between BSMV:00*‐* and BSMV:*TaERF1L‐* silenced plants under the N and NHN treatments at 120 hptt. However, the number of pustules per unit leaf area was significantly greater (*p* < 0.05) in the *TaERF1L*‐silenced leaves compared to BSMV:00 leaves under the NHN treatment (Figure S7c,d). These results indicated that silencing *TaERF1L* reduced HTAS resistance to Pst in XY6.

## 3 Discussion

Temperature‐sensitive resistance to pathogens widely exists in many plant species, including in wheat and related grass species against Pst. Qayoum and Line (1985) were the first to propose the concept of wheat high‐temperature resistance to Pst, suggesting that as temperature rises, wheat can activate resistance against the pathogen. Xiaoyan 6 (XY6), a high‐yield and high‐quality wheat cultivar possessing typical HTAS resistance, has maintained strong resistance to stripe rust since its release. Previous transcriptomics studies proved that multiple receptor‐like kinases (RLKs) (Shi et al. 2023; Wang et al. 2021), *R* genes (Wang et al. 2019; Hu et al. 2021), Pst effectors (Hu, Su, et al. 2022) and WRKY TFs (Wang, Tao, Wang, An, et al. 2017; Wang, Tao, Wang, Tian, et al. 2017) are involved in regulating HTAS resistance in XY6. In the present study, both Pst infection and the relatively high temperature treatment led to an up‐regulated expression level of the *TaRABH1bL* gene, which significantly exceeded those treated under normal temperature conditions with Pst infection (Figure 1b). VIGS‐mediated *TaRABH1bL* gene silencing significantly reduced the HTAS resistance in XY6 (Figure 2). These findings suggested that TaRABH1bL may function as a positive regulator in mediating the HTAS resistance in XY6 to Pst under fluctuating temperature conditions.

Rab GTPases have been shown to play critical roles in plant biotic and abiotic stress tolerance. Under biotic stress conditions, *Phytophthora infestans* core RXLR effector Pi17063 functions as the specific GTPase‐activating protein (GAP), suppressing plant immunity by targeting the host plasma membrane and NbRab‐G3 proteins in *N. benthamiana* (Liu and Ding 2025). Knocking out *RabE1a* in tomato inhibits tomato brown rugose fruit virus (ToBRFV), tobacco mosaic virus (TMV), tomato mosaic virus (ToMV) and tomato mottle mosaic virus (ToMMV) infection. Additionally, RabE1a interacts with ToBRFV movement protein and the exocyst subunit SEC10b to positively regulate movement protein transport to the plasma membrane and intracellular movement of ToBRFV (Ma et al. 2025). For abiotic stress regulations, the expression level of *OsRab6a* is rapidly and transiently up‐regulated under low iron conditions; overexpression of *OsRab6a* confers greater tolerance to iron deficiency, higher seedling height and greater biomass than that in RNAi and wild‐type rice plants (Yang and Zhang 2016). Furthermore, cold stress strongly induces *OsRab6a* expression. OsRab6a acts upstream of and interacts with a cold‐resistance protein OsRAN2. Additionally, OsRab6a up‐regulates *OsPYL3* expression to modulate abscisic acid (ABA) signalling during cold stress (Li et al. 2025). In XY6, *TaRABH1bL* also functions as a regulator in response to biotic and abiotic stresses, corresponding to relatively high temperature treatment and Pst inoculation to activate HTAS resistance.

**FIGURE 8:**
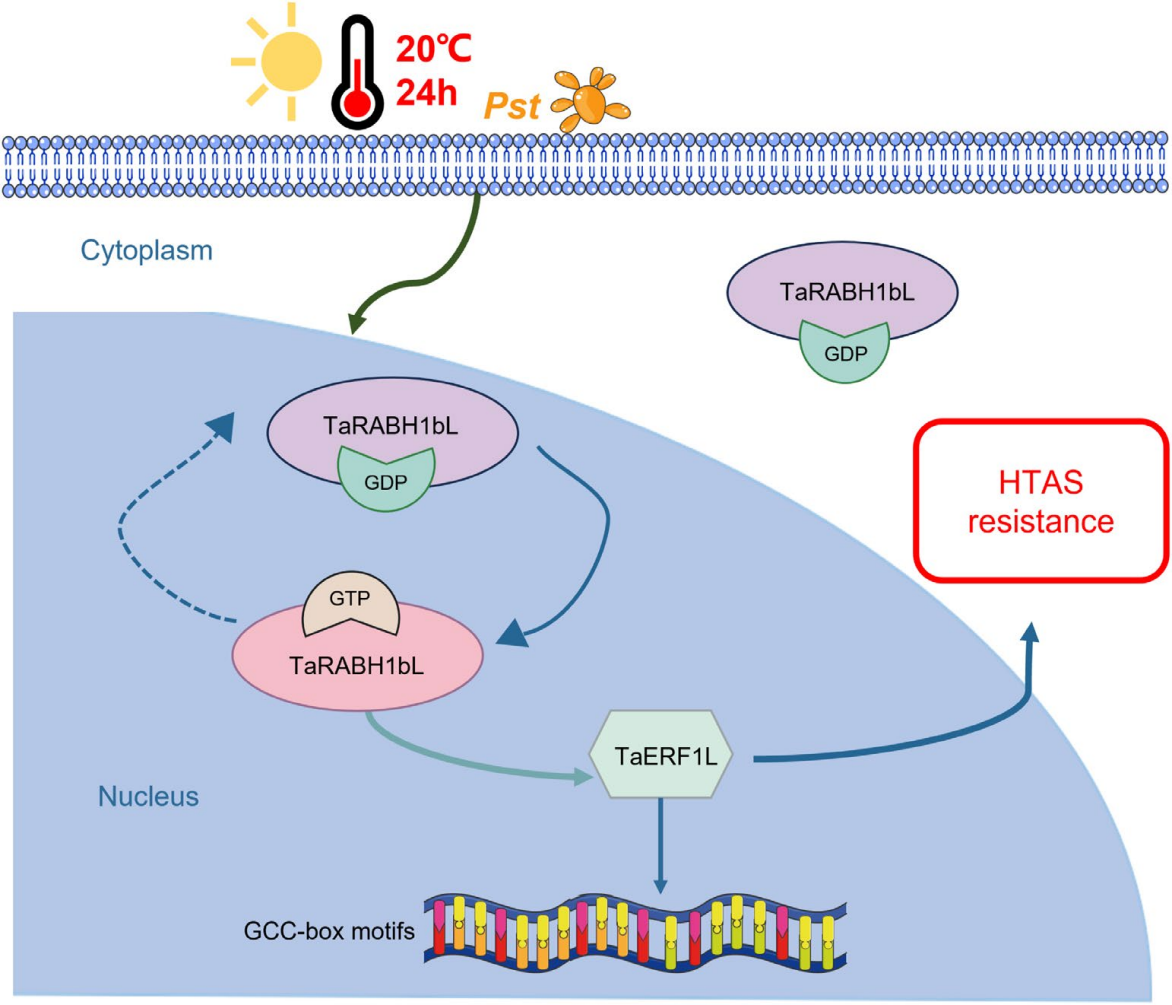
A working model of small GTPase gene *TaRABH1bL* in high‐temperature all‐stage (HTAS) resistance against *Puccina striiformis* f. sp. *tritici* (Pst) in wheat Xiaoyan 6 (XY6) under a relatively high temperature (20°C). Upon Pst infection and the relatively high temperature treatment, TaRABH1bL simultaneously senses these biotic and abiotic stress signals and interacts with the transcription factor TaERF1L in nuclei, specifically in its active (GTP‐binding) state. This interaction transmits immune signals and regulates the HTAS resistance in XY6. Solid lines represent well‐studied results, while the dotted lines represent our hypothesis.

As stress‐responsive TFs, ERF TFs play a crucial role in response to biotic stress. VaERF16 participates in disease resistance to *Botrytis cinerea* by interacting with another stress‐responsive TF VaMYB306, forming a complex and binding to the GCC box of the defence‐related gene *VaPDF1.2* promoter in grape (Zhu et al. 2022). Under abiotic stress conditions, ERF also plays an important regulatory role. In soybean, GmERF1 negatively regulates low phosphorus tolerance by interacting with the TF GmWRKY6 to mediate 1‐amino‐cyclopropane‐1‐carboxylic acid (ACC) concentration and inhibit downstream gene expression including *GmPT5*, *GmPT7* and *GmPT8* (Wang et al. 2023). In the present study, TaERF1L was found to interact with TaRABH1bL in vivo and in vitro (Figure 4). Silencing the *TaERF1L* gene resulted in reduced resistance to Pst under relatively high temperature conditions. These results indicated that TaERF1L positively regulates both biotic and abiotic signals transmitted from TaRABH1bL, thereby initiating the HTAS resistance in XY6 (Figure 7). Furthermore, TFs primarily activate or inhibit gene expression by binding to their promoter regions. *TaPIE1* activates downstream defence‐ and stress‐related genes including *PR10*, *ICE1*, *POX2* and *P5CR* by binding to their GCC‐box elements, thereby enhancing resistance against *Rhizoctonia cerealis* and cold stress tolerance (Zhu et al. 2014). In this study, Y1H and dual‐LUC assays demonstrated that TaERF1L bound to and repressed the GCC‐box *cis*‐elements (Figure 6a–c). However, further investigation is required to identify the specific susceptible‐ and temperature‐sensitive genes containing GCC‐box motifs that are directly regulated by TaERF1L. Small GTPases cycle between active (GTP‐binding) and inactive (GDP‐binding) states on receiving upstream signals. Previous studies indicated that small GTPases in different states modulate various physiological and biochemical processes in plants (Bourne et al. 1991; Valencia et al. 1991; Nielsen 2020). Overexpression of the constitutively inactive‐state mutant of CaRop1 (DN‐CaRop1), a Rho subfamily GTPase, substantially enhances resistance to *Ralstonia solanacearum* and aphid infection in tobacco. In contrast, transgenic tobacco plants with the active‐state mutant CA‐CaRop1 show enhanced susceptibility to *R. solanacearum* and aphid infection with down‐regulated expression of PTI‐related genes (Qiu et al. 2016). To determine the effects of different states of TaRABH1bL on its function, the active‐state mutant (*TaRABH1bL* ^*Q69L*^) and inactive‐state mutant (*TaRABH1bL* ^*N122I*^) of the *TaRABH1bL* gene were generated. Their functions were evaluated using Y2H, pull‐down and BiFC assays. As shown in Figure 5d–g, TaERF1L exclusively interacted with TaRABH1bL in the GTP‐binding state for immune signal transduction. Additionally, previous studies have demonstrated that different active states can influence the subcellular localisation of small GTPases. In *Arabidopsis*, AtRabD2b and its active‐state mutant AtRabD2b^Q67L^ are localised in Golgi stacks while its inactive‐state mutant AtRabD2b^N121I^ is dispersed throughout the cytoplasm (Wang et al. 2012). However, subcellular localisation assays of different active states of TaRABH1bL conducted in *N. benthamiana* leaves and wheat protoplasts revealed that both TaRABH1bL and its mutants (TaRABH1bL^Q69L/N122I^) were localised in the cytoplasm and nuclei (Figure 5a–c). Based on the dual‐LUC assays in *N. benthamiana* leaves, the inhibitory effect of TaERF1L on the GCC‐box motif was strengthened by the exclusive interaction between TaRABH1bL^Q69L^ and TaERF1L (Figure 6d). In conclusion, dual signals of Pst infection and the relatively high temperature treatment altered the active states of TaRABH1bL and interfered with its interaction with TaERF1L without affecting the subcellular localisation; the GTP‐binding state of TaRABH1bL might activate HTAS resistance through enhancing the inhibition of TaERF1L on the expression of particular genes with the GCC‐box motif in their promoters. In this study, we proposed a new molecular regulatory model for a small GTPase involved in HTAS resistance. As the ‘molecular switch’, TaRABH1bL resides in both cytoplasm and nuclei in its inactive (GDP‐binding) state. However, upon receiving the dual signals of biotic (Pst infection) and abiotic (the relatively high temperature treatment) stress, only the nuclei‐located TaRABH1bL converted into the active (GTP‐binding) state and interacted with the TF TaERF1L. The active state of TaRABH1bL might increase the suppression of TaERF1L on its downstream susceptible or temperature‐sensitive genes containing the GCC‐box motif, thereby activating HTAS resistance to Pst in XY6 (Figure 8). The unique ability of TaRABH1bL to integrate both Pst infection and temperature signalling through GTP‐binding‐dependent interaction with TaERF1L provides a novel molecular mechanism for breeding climate‐resilient wheat cultivars. By engineering the GTP‐binding domain with other temperature‐responsive resistance genes through marker‐assisted selection, breeders could develop cultivars maintaining HTAS resistance under rising global temperatures.

## 4 Experimental Procedures

### 4.1 Plant Materials and Temperature Treatments

Wheat ( *Triticum aestivum* ) cultivars Xiaoyan 6 (XY6) with HTAS resistance and Mingxian 169 (MX169) susceptible to stripe rust, along with *N. benthamiana* and Pst CYR32 (Chinese yellow rust 32) were obtained from the College of Plant Protection, Northwest A&F University, Yangling, Shaanxi, China. At the two‐leaf stage, wheat plants were inoculated with CYR32 and placed in a dew chamber at 10°C without light for 24 h. The inoculated plants were grown under a 16‐h light (15°C)/8‐h dark (10°C) cycle, and divided into two groups before temperature treatments: (i) normal temperature (N) treatment: the plants were maintained at 15°C for the entire duration of the experiment; and (ii) normal‐high‐normal temperature (NHN) treatment, also called the relatively high temperature treatment: the plants were kept at 15°C until 192 hpi, then transferred to a 20°C growth chamber for 24 h before returning to the 15°C growth chamber. For each temperature condition, non‐inoculated plants grown under the same conditions served as controls (mock).

### 4.2 Cloning and Bioinformatics Analyses of *TaRABH1bL* and *TaERF1L*

Total RNA of XY6 under different treatments was extracted according to the instructions of the SV Total RNA Isolation System (Promega). First‐strand cDNA was synthesised using a PrimeScript RT reagent Kit (TaKaRa). Primers for amplifying the full length of *TaRABH1bL* and its mutated variants *TaRABH1bL* ^*Q69L/N122I*^ and *TaERF1L* were designed using Premier 6.0 (Table S1) from the wheat UGRI genome database (https://urgi.versailles.inra.fr). The amino acid sequences of TaRABH1bL and its homologues from other plant species were obtained from the National Center for Biotechnology Information (NCBI) database (https://www.ncbi.nlm.nih.gov/), and DNAMAN 6.0 was used for multisequence alignment.

### 4.3 Tissue‐Specific Expression and Transcription Level Analyses of *TaRABH1bL* in Response to Pst Infection and Temperature Treatments

The expression level of *TaRABH1bL* in leaves, stems and roots of XY6 was quantified by RT‐qPCR. To investigate the transcript profiles of *TaRABH1bL* in relation to HTAS resistance, wheat samples were collected at 0, 72, 192, 204, 216, 240, 264 and 312 hpi. The wheat 26S proteasome regulatory subunit (*Ta26S*) gene was used as the reference for the RT‐qPCR assay (Wang, Tao, Wang, An, et al. 2017). Every sampling included three biological replications. The 2^−ΔΔ*C*t^ method was used for RT‐qPCR analyses.

### 4.4 Subcellular Localisation

For subcellular localisation in *N. benthamiana* leaves, *Agrobacterium tumefaciens* GV3101 (Weidibio) harbouring recombinant vectors TaRABH1bL‐GFP, TaRABH1bL^Q69L^‐GFP, TaRABH1bL^N122I^‐GFP and TaERF1L‐RFP were infiltrated into leaves. To confirm the accurate localisation of TaRABH1bL, 4′,6‐diamidino‐2‐phenylindole (DAPI) staining was used to indicate the nuclei and TaWPI6‐mCherry was used as a plasma membrane marker (Xu et al. 2022). Different components of proteins were extracted using the Membrane and Cytosol Protein Extraction Kit or Nuclear and Cytoplasmic Protein Extraction Kit (Beyotime). Membranal, cytoplasmic and nuclear proteins were detected by western blot using the anti‐GFP antibody (Beyotime). To test the subcellular localisation in wheat protoplasts, TaRABH1bL‐GFP, TaRABH1bL^Q69L^‐GFP and TaRABH1bL^N122I^‐GFP recombinant plasmids were transformed into XY6 seedling leaf cells using the Wheat Protoplast Preparation and Transformation Kit (Coolaber). The fluorescence signals were observed under the confocal microscope FV3000 (Olympus). Total protein was extracted from wheat protoplasts and detected by western blot using the anti‐GFP antibody (Beyotime).

### 4.5 BSMV‐Mediated *TaRABH1bL* and *TaERF1L* Gene Silencing

The barley stripe mosaic virus (BSMV) inoculum system was combined with BSMV:*α*, BSMV:*β* and BSMV:*TaRABH1bL* or BSMV:*TaERF1L* plasmids, which were transcribed in vitro using the RiboMAX large‐scale RNA production systems (Promega). Transcribed RNAs were combined with FES buffer and inoculated on XY6 wheat leaves. The BSMV:*TaPDS* and BSMV:*γ* empty vector (BSMV:00) were used as positive and negative controls, respectively. Wheat seedlings inoculated with the 1× FES buffer were used as the mock control (mock). Once photobleaching was observed on the BSMV:*TaPDS*‐infected leaves, the fourth leaves of XY6 were inoculated with Pst CYR32 followed by N and NHN temperature treatments. The silencing efficiency of *TaRABH1BL* and *TaERF1L* was assessed at 0, 12, 48, 72 and 120 hpi. Seedling samples were collected at 0, 12, 24, 48, 72 and 120 hptt to further evaluate the expression level of *TaRABH1bL* and *TaERF1L* under different temperature treatments. Additionally, the expression level of resistance genes *TaPR1* and *TaPR2* was assessed at 0, 24, 48 and 120 hptt in *TaRABH1bL*‐ and *TaERF1L*‐silenced plants. For histological observations, samples were collected at 24, 48 and 120 hpi for counting the number of haustorial mother cells and measuring the hyphal length of Pst. At 120 hptt, wheat leaves were collected to measure the linear length of uredinia and the number of pustules of Pst per unit leaf area. Infection types (ITs) were recorded at 14 dpi on a 0 to 9 scale (0–3: highly resistant; 4–6: moderately resistant; 7–9: highly susceptible) (Chen et al. 2021). The detailed protocols for the histological observations were performed as previously described (Wang, Tao, Wang, An, et al. 2017).

### 4.6 Y2H, BiFC, Split‐LUC Complementation, Co‐IP and Pull‐Down Assays

For Y2H assays, the open reading frame (ORF) region of the *TaRABH1bL* gene was cloned into the pGBKT7 (BD) vector firstly to exclude its autoactivation and toxicity in yeast cells, and then the TaRABH1bL fusion protein was used as bait to identify target proteins. To further confirm the interactions between TaRABH1bL and its potential target proteins, the ORF region of the *TaERF1L* gene was cloned into the pGADT7 (AD) vector. Yeast strains Y2HGold (Weidibio) containing BD‐TaRABH1bL and AD‐TaERF1L recombinant plasmids were grown on SD/−Trp/−Leu medium (double dropout, DDO) (Coolaber). Mature colonies were transferred onto SD/−Trp/−Leu/−His/+X‐α‐Gal medium (triple dropout, TDO/X). The coding sequences of *TaRABH1bL* and *TaERF1L* were respectively cloned into pER8‐Ne/CeYFP vectors for BiFC assay, the fusion constructs were co‐transformed into *A. tumefaciens* GV3101 (Weidibio) and infiltrated into *N. benthamiana* tobacco leaves. The yellow fluorescence was observed using an FV3000 confocal laser scanning microscope. For split‐LUC complementation assay, the coding sequences of *TaRABH1bL* and *TaERF1L* were respectively inserted into c/nLuc vectors. Leaves were placed in the dark for 48 h and then sprayed with 1 mM D‐luciferin (Solarbio). After keeping in the dark for 5 min, the fluorescent signals were recorded using the PlantView100 Plant in vivo imaging system (Photon Technology). A co‐IP assay between TaRABH1bL‐GFP and TaERF1L‐FLAG was performed using *N. benthamiana* leaves. Total protein was extracted and immunoprecipitated with GFP‐Trap Agarose (ChromoTek) according to the manufacturer's protocol. The immunoprecipitated proteins were detected by immunoblotting with anti‐FLAG and anti‐GFP antibodies (Beyotime). For in vitro pull‐down assays, the recombinant plasmids TaRABH1bL‐His and TaERF1L‐GST were expressed in *Escherichia coli* BL21 cells (Weidibio). The pull‐down assay was performed using the GST Protein Interaction Pull‐Down Kit (Pierce, Thermo Fisher Scientific) according to the manual instructions. The recombinant proteins were separated by 10% SDS‐PAGE and immunoblotting with corresponding antibodies. Three biological replicates were performed in Y2H, BiFC, split‐LUC complementation, co‐IP and pull‐down assays.

### 4.7 Y1H and Dual‐LUC Assays

For Y1H assay, the pmotif (GCC‐box)‐AbAi and AD‐TaERF1L were co‐transformed into yeast strain Y1HGold (Weidibio) and cultured on SD/−Leu medium with 200 ng/mL AbA added. Yeast cells harbouring p53‐AbAi + AD‐p53 and p53‐AbAi + AD‐53/p53‐AbAi + AD served as positive and negative controls, respectively. For dual‐LUC assay, the 5 × GCC‐box‐mini35S was inserted into pGreenII‐0800‐LUC to obtain the reporter, and the coding sequence of *TaERF1L* was inserted into the pGreenII 62‐SK vector to serve as the effector. *A. tumefaciens* strains harbouring the different constructs were co‐infiltrated into the *N. benthamiana* leaves, leaves were sprayed with 1 mM D‐luciferin and then the PlantView100 Plant in vivo imaging system was used to detect luciferase luminescence. Three biological replicates were performed per assay.

### 4.8 Statistical Analyses

Data are presented as the standard error of the mean (SEM) (*n* = 3). For multiple comparisons, Duncan's multiple range test was conducted and the significance of the test was assessed at a probability value of *p* < 0.05. For two‐sample comparison, a Student's *t* test was employed and the significance of the test was assessed at a probability value of *p* < 0.05. GraphPad Prism 8.0 has been used for all analyses.

## Author Contributions

**Yifeng Shi:** writing – review and editing, writing – original draft, methodology, formal analysis, software, data curation, validation, investigation, visualization. **Xiyue Bao:** writing – review and editing, software, data curation, validation, formal analysis, investigation, visualization. **Hai Li:** software, data curation, validation, investigation, formal analysis. **Yuxiang Li:** writing – review and editing, resources, supervision, data curation, project administration, validation. **Xianming Chen:** writing – review and editing, project administration, resources, supervision, data curation, validation. **Xiaoping Hu:** writing – review and editing, conceptualization, funding acquisition, project administration, resources, supervision, data curation, validation.

## Conflicts of Interest

The authors declare no conflicts of interest.

## Supporting information


**Figure S1.** Three small GTPase genes were identified via transcriptomics analysis of wheat cultivar Xiaoyan 6 (XY6). TaRABH1bL: Ras‐related protein RABH1b‐like; TaRABC1L: Ras‐related protein RABC1‐like; TaGSP1: GTP‐binding nuclear protein gsp1/Ran.

**Figure S2.** Sequence alignment and domain architecture prediction of TaRABH1bL. (a) Predicted domain architecture of TaRABH1bL. The domain architecture prediction was performed using SMART analysis. (b) Gene sequence of *TaRABH1bL* with three copies (*TaRABH1bL*‐*4A*, *TaRABH1bL*‐*4B* and *TaRABH1bL*‐*4D*) were downloaded from the wheat UGRI genome database. The red line box indicated the fragment for *TaRABH1bL* gene silencing while the green line box represented the *TaRABH1bL* fragment for qRT‐PCR analyses.

**Figure S3.** The expression levels of *TaRABH1bL* in response to high‐temperature all‐stage (HTAS) resistance in susceptible wheat cultivar Mingxian 169 (MX169). N mock: normal temperature (15°C) without Pst inoculation; N: normal temperature (15°C) with Pst inoculation; NHN mock: high‐temperature (20°C) for 24 h exposure at 192 h post inoculation (hpi) and then transferred to 15°C without Pst inoculation; NHN: high‐temperature (20°C) for 24 h at 192 hpi and then transferred to 15°C with Pst inoculation. Duncan’s multiple range test (*p* < 0.05) was conducted to test statistical significance. Three independent biological replicates were conducted for the qRT‐PCR analyses.

**Figure S4.** Autoactivation and toxicity detection of pGBKT7 (BD)‐TaRABH1bL. Yeast cells harbouring BD‐53 + pGADT7 (AD)‐T and BD‐Lam + AD‐T plasmids grown on SD/−Trp/−Leu/5‐bromo‐4‐chloro‐3‐indoxyl‐α‐D‐galactopyranoside (X‐α‐gal) (Double Dropout, DDO/X) medium were used as positive and negative controls, respectively. Yeast cells harbouring BD‐TaRABH1bL were spread on SD/−Trp (SDO), SD/−Trp/X‐a‐Gal (SDO/X) and SD/−Trp/X‐a‐Gal/AbA (SDO/X/A) plates, respectively.

**Figure S5.** Amino acid sequence alignment of TaRABH1bL and its homologues from various species. *Lolium rigidum* (Lr); *Brachypodium distachyon* (Bd); *Panicum hallii* (Ph); *Oryza sativa* (Os); *Panicum virgatum* (Pv); *Setaria italica* (Si); *Panicum miliaceum* (Pm); *Sorghum bicolor* (Sb); and *Zea mays* (Zm). G1‐G5 motifs: the unique motifs of Rab proteins.

**Figure S6.** Subcellular co‐localisations of TaRABH1bL, TaRABH1bL^Q69L/N122I^ and TaERF1L in nuclei of tobacco leaves. Green fluorescence signal represented the localisation of TaRABH1bL‐GFP, TaRABH1bL^Q69L/N122I^‐GFP and GFP proteins, red fluorescence represented the localisation of TaERF1L‐RFP protein and the yellow overlapping fluorescence signal represented co‐localisation of TaRABH1bL, TaRABH1bL^Q69L/N122I^ with TaERF1L proteins. Bars = 50 μm.

**Figure S7.** Hyphal length (a) and the number of haustorial mother cells (b) were assessed at 24, 48 and 120 hpi. Student’s *t*‐test (*p* < 0.05) was conducted to test for statistical significance; Pustules number per unit leaf area (c) and length of uredinia (d) in *TaERF1L*‐silenced and non‐silenced wheat plants were calculated at 120 hptt. Student’s *t*‐test (*p* < 0.05) was conducted to test for statistical significance.

**Table S1.** Primers used in this research.

## Data Availability

The data supporting the findings of this study are available from the corresponding author upon reasonable request.
