# Supplementary figures and images for: Wheat Small GTPase Gene TaRABH1bL Is Involved in High‐Temperature All‐Stage Resistance to Puccinia striiformis f. sp. tritici
Source: Mol Plant Pathol. 2025 Aug 7;26(8):e70132. doi: 10.1111/mpp.70132 (PMC12330980; doi:10.1111/mpp.70132)

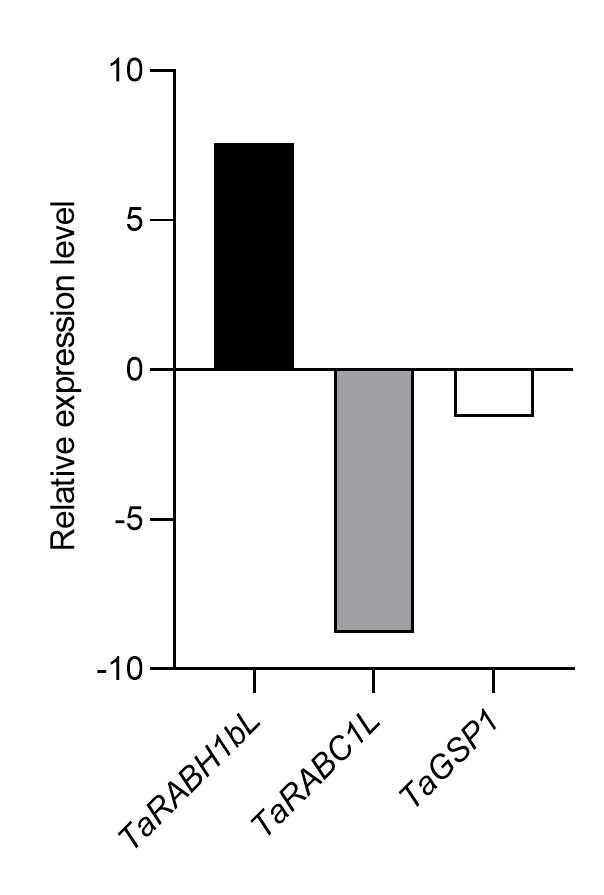

Supplement: Supplementary file 1 — Figure S1. Three small GTPase genes were identified via transcriptomics analysis of wheat cultivar Xiaoyan 6 (XY6). TaRABH1bL: Ras‐related protein RABH1b‐like; TaRABC1L: Ras‐related protein RABC1‐like; TaGSP1: GTP‐binding nuclear protein gsp1/Ran. [file MPP-26-e70132-s007.tif]

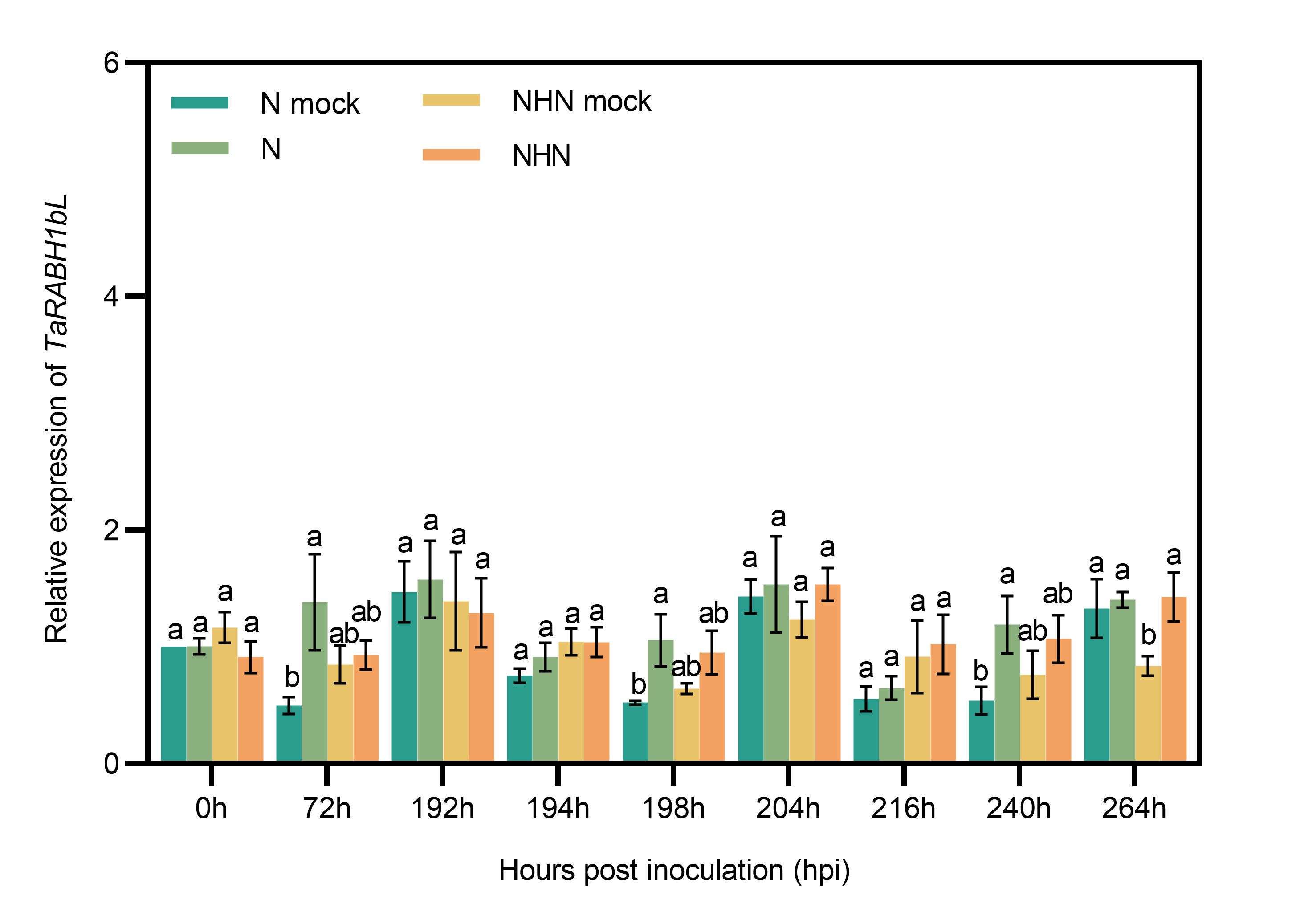

Supplement: Supplementary file 3 — Figure S3. The expression levels of TaRABH1bL in response to high‐temperature all‐stage (HTAS) resistance in susceptible wheat cultivar Mingxian 169 (MX169). N mock: normal temperature (15°C) without Pst inoculation; N: normal temperature (15°C) with Pst inoculation; NHN mock: high‐temperature (20°C) for 24 h exposure at 192 h post inoculation (hpi) and then transferred to 15°C without Pst inoculation; NHN: high‐temperature (20°C) for 24 h at 192 hpi and then transferred to 15°C with Pst inoculation. Duncan’s multiple range test (p < 0.05) was conducted to test statistical significance. Three independent biological replicates were conducted for the qRT‐PCR analyses. [file MPP-26-e70132-s008.tif]

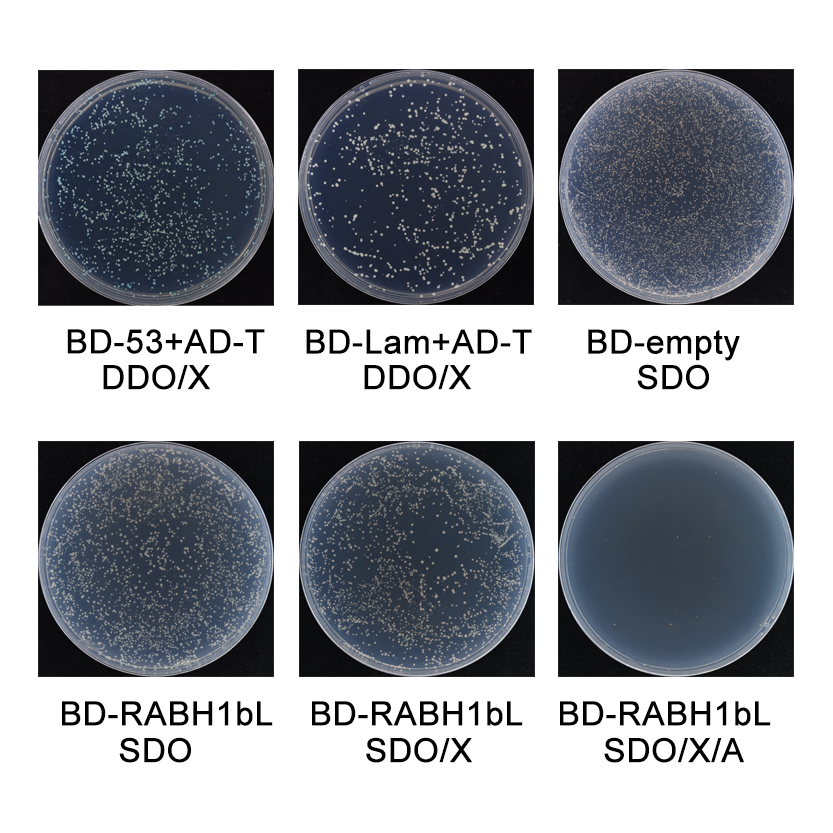

Supplement: Supplementary file 4 — Figure S4. Autoactivation and toxicity detection of pGBKT7 (BD)‐TaRABH1bL. Yeast cells harbouring BD‐53 + pGADT7 (AD)‐T and BD‐Lam + AD‐T plasmids grown on SD/−Trp/−Leu/5‐bromo‐4‐chloro‐3‐indoxyl‐α‐D‐galactopyranoside (X‐α‐gal) (Double Dropout, DDO/X) medium were used as positive and negative controls, respectively. Yeast cells harbouring BD‐TaRABH1bL were spread on SD/−Trp (SDO), SD/−Trp/X‐a‐Gal (SDO/X) and SD/−Trp/X‐a‐Gal/AbA (SDO/X/A) plates, respectively. [file MPP-26-e70132-s003.tif]

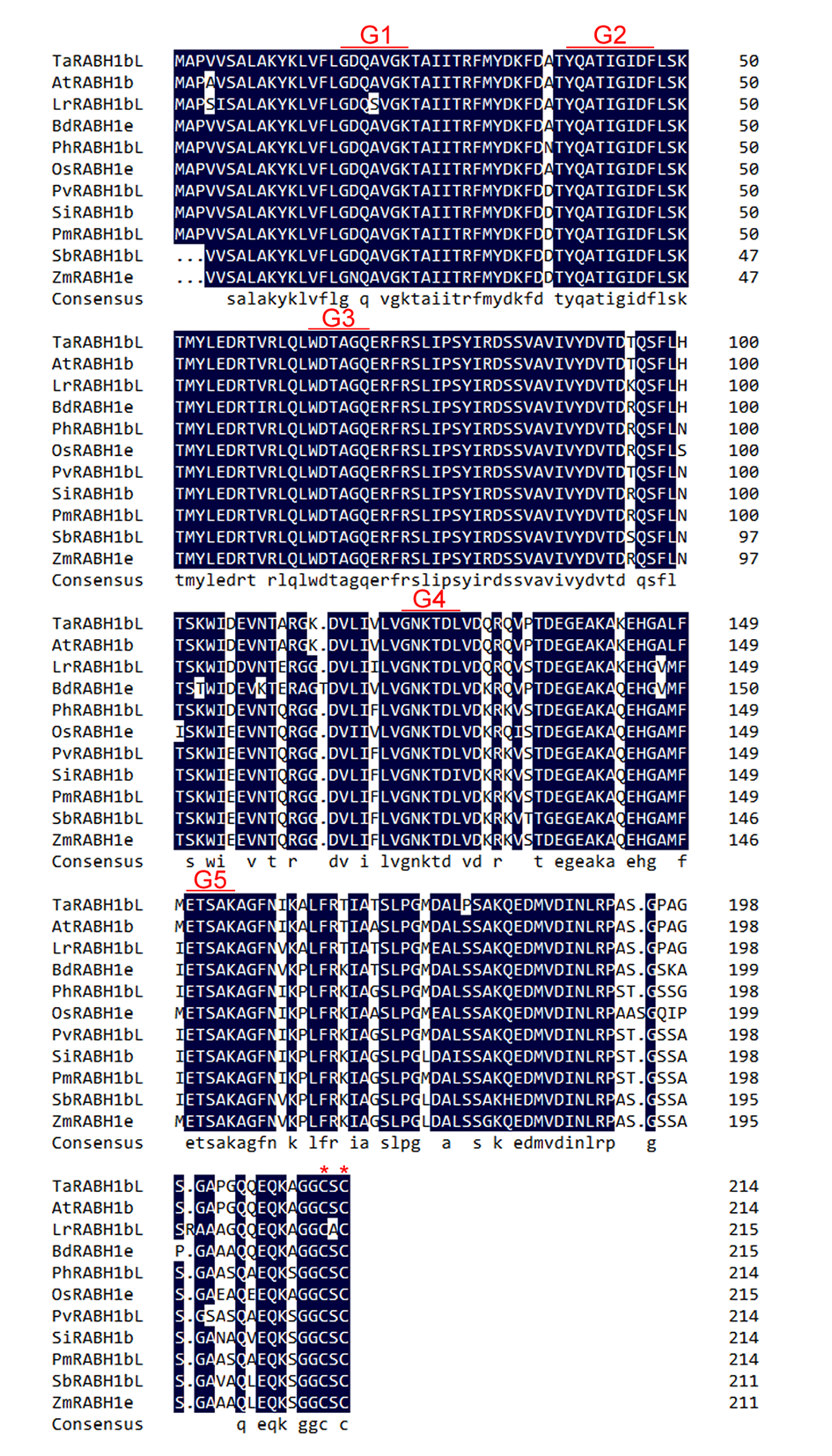

Supplement: Supplementary file 5 — Figure S5. Amino acid sequence alignment of TaRABH1bL and its homologues from various species. Lolium rigidum (Lr); Brachypodium distachyon (Bd); Panicum hallii (Ph); Oryza sativa (Os); Panicum virgatum (Pv); Setaria italica (Si); Panicum miliaceum (Pm); Sorghum bicolor (Sb); and Zea mays (Zm). G1‐G5 motifs: the unique motifs of Rab proteins. [file MPP-26-e70132-s006.tif]

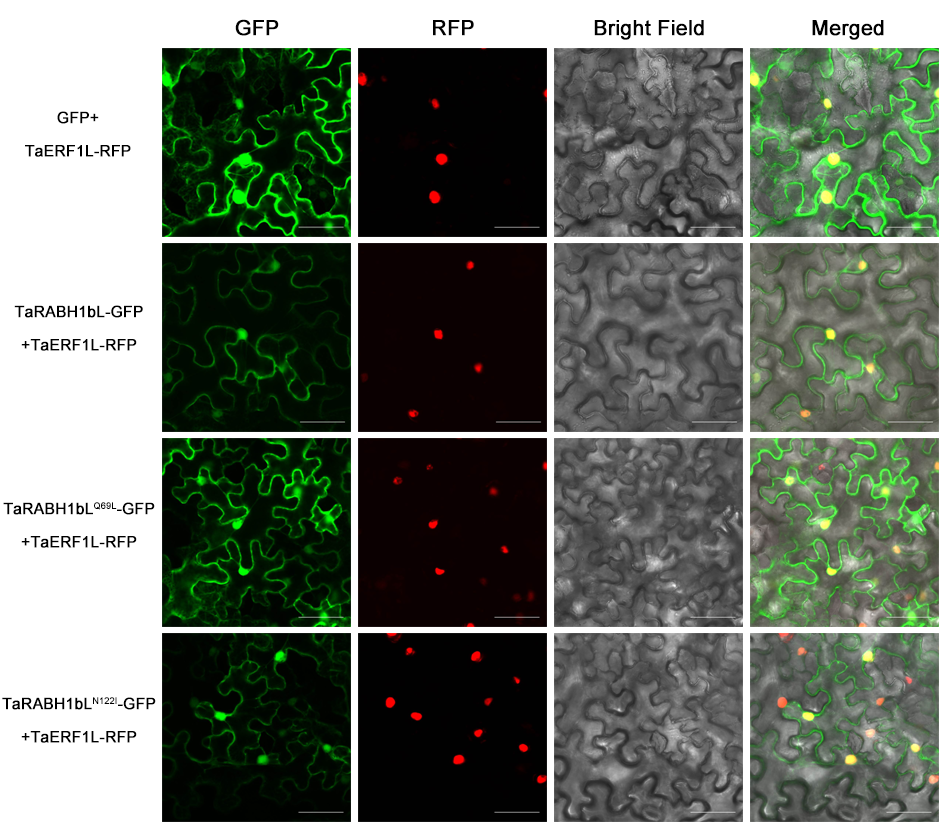

Supplement: Supplementary file 6 — Figure S6. Subcellular co‐localisations of TaRABH1bL, TaRABH1bLQ69L/N122I and TaERF1L in nuclei of tobacco leaves. Green fluorescence signal represented the localisation of TaRABH1bL‐GFP, TaRABH1bLQ69L/N122I‐GFP and GFP proteins, red fluorescence represented the localisation of TaERF1L‐RFP protein and the yellow overlapping fluorescence signal represented co‐localisation of TaRABH1bL, TaRABH1bLQ69L/N122I with TaERF1L proteins. Bars = 50 μm. [file MPP-26-e70132-s005.tif]

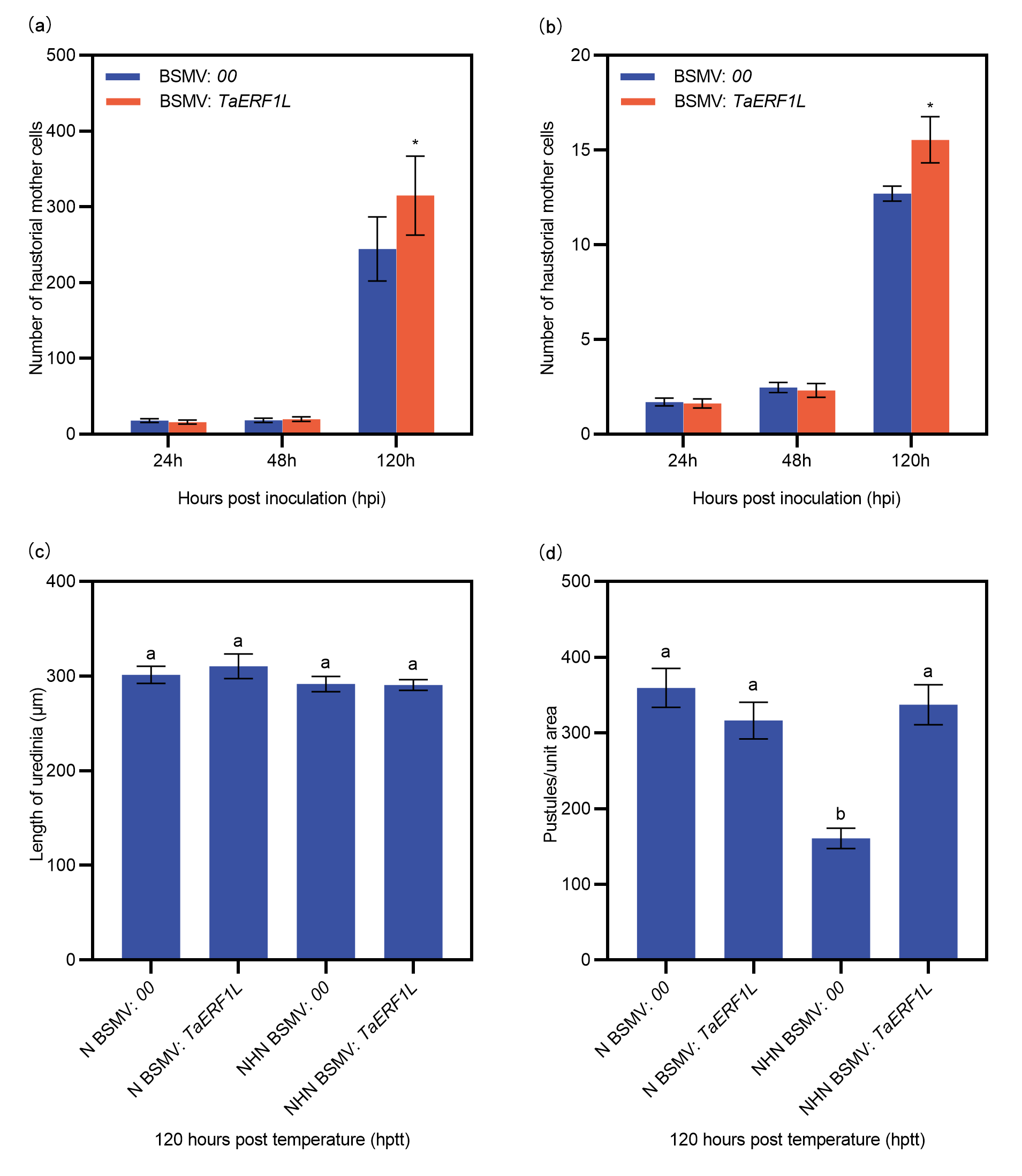

Supplement: Supplementary file 7 — Figure S7. Hyphal length (a) and the number of haustorial mother cells (b) were assessed at 24, 48 and 120 hpi. Student’s t‐test (p < 0.05) was conducted to test for statistical significance; Pustules number per unit leaf area (c) and length of uredinia (d) in TaERF1L‐silenced and non‐silenced wheat plants were calculated at 120 hptt. Student’s t‐test (p < 0.05) was conducted to test for statistical significance. [file MPP-26-e70132-s004.tif]
