# Supplementary material for: Wheat Small GTPase Gene TaRABH1bL Is Involved in High‐Temperature All‐Stage Resistance to Puccinia striiformis f. sp. tritici
Source: Mol Plant Pathol. 2025 Aug 7;26(8):e70132. doi: 10.1111/mpp.70132 (PMC12330980; doi:10.1111/mpp.70132)
Supplement: Supplementary file 8 — Table S1. Primers used in this research. [file MPP-26-e70132-s002.docx]

| **Primers used in this study** |  |  |
| --- | --- | --- |
| **Primer name** | **Primer sequence (5′-3′)** | **Description** |
| VIGS-TaRABH1bL-F | GCGGCCGCAAGACCATGTACCTCG | Gene silencing |
| VIGS-TaRABH1bL-R | TTAATTAAATGTATGCAGAAACGA |  |
| VIGS-TaERF1L-F | GCGGCCGCCCGGGGCGCGCACGCCCT | Gene silencing |
| VIGS-TaERF1L-R | TTAATTAACGGGCGGCGGCTCCCGCT |  |
| qTaRABH1bL-F | GCCAAGTACAAGCTGGTGTTCC | qRT-PCR |
| qTaRABH1bL-R | ATCCCGATGGTCGCCTGGTA |  |
| qTaERF1L-F | GTTGGAACTGGTGCTCTG | qRT-PCR |
| qTaERF1L-R | GACCGCTTCTTTCGTTTC |  |
| qTa26S-F | GCTGGCTCGTTCAACTGATG | qRT-PCR |
| qTa26S-R | GGACCAAGCGTTCTGATTACTC |  |
| pGBKT7-TaRABH1bL-F | GAATTCATGGCGCCGGTGGTGTCG | Yeast-two-hybrid |
| pGBKT7-TaRABH1bL-R | GGATCCTCAGCAAGAGCATCCTCC |  |
| pGADT7-TaERF1L-F | GAATTCATGGGGCCCAACCCTAGC | Yeast-two-hybrid |
| pGADT7-TaERF1L-R | GAATTCTCAGGTCCCGCCAACTTC |  |
| TaRABH1bL-YFP^C^-F | CTCGAGATGGCGCCGGTGGTGTCG | BiFC |
| TaRABH1bL-YFP^C^-R | CCTAGGGCAAGAGCATCCTCCTGC |  |
| TaERF1L-YFP^N^-F | CCTAGGATGGAGTCGGCGGCCGAG | BiFC |
| TaERF1L-YFP^N^-R | ACTAGTGGTCCCGCCAACTTCCTC |  |
| TaRABH1bL-GFP-F | GGGCCCATGGCGCCGGTGGTGTCGGC | Subcellular localization |
| TaRABH1bL-GFP-R | GGGCCCTCAGCAAGAGCATCCTCCTG |  |
| TaERF1L-GFP-F | GGGCCCATGGAGTCGGCGGCCGAG | Subcellular localization |
| TaERF1L-GFP-R | GGGCCCTCAGGTCCCGCCAACTTC |  |
| TaERF1L-RFP-F | GGGCCCATGGAGTCGGCGGCCGAG | Subcellular localization |
| TaERF1L-RFP-R | GGGCCCGGTCCCGCCAACTTCCTCG |  |
| p16318GFP-TaRABH1bL-F | GGATCCATGGCGCCGGTGGTGTCG | Subcellular localization |
| p16318GFP-TaRABH1bL-R | GGATCCGCAAGAGCATCCTCCTGC |  |
| p16318GFP-TaERF1L-F | GGATCCATGGGGCCCAACCCTAGC | Subcellular localization |
| p16318GFP-TaERF1L-R | GGATCCGGTCCCGCCAACTTCCTC |  |
| pET28a-TaRABH1bL-F | GAATTCATGGCGCCGGTGGTGTCG | Pull-down |
| pET28a-TaRABH1bL-R | GAATTCGCAAGAGCATCCTCCTGC |  |
| pGEX4T-TaERF1-F | GAATTCATGGAGTCGGCGGCCGAG | Pull-down |
| pGEX4T-TaERF1-R | GTCGACGGTCCCGCCAACTTCCTC |  |
| TaERF1-nLuc-F | GGTACCATGGAGTCGGCGGCCGAG | split-LUC complementation assay |
| TaERF1-nLuc-R | GTCGACGGTCCCGCCAACTTCCTC |  |
| TaRABH1bL-cLuc-F | GGTACCATGGCGCCGGTGGTGTCG | split-LUC complementation assay |
| TaRABH1bL-cLuc-R | GTCGACGCAAGAGCATCCTCCTGC |  |
| 62sk-TaERF-F | TGGATCC ATGGGGCCCAACCCTAG | dual-LUC complementation assay |
| 62sk-TaERF-R | GAATTC TCAGGTCCCGCCAACTTCC |  |
| 0800-5×GCC-F | GTCGACAGCCGCCAGCCGCCAGCCGCCAGCCGCCAGCCGCCGCAAGACCCTTCCTCTATA | dual-LUC complementation assay |
| 0800-5×GCC-R | GGATCCTCCTCTCCAAATGAAATGAACTTCCTTATATAGAGGAAGGGTCTTGC |  |
| AD-TaERF1L-F | GAATTC ATGGGGCCCAACCCTAG | Yeast-one-hybrid |
| AD-TaERF1L-R | GAATTCTCAGGTCCCGCCAACTTCC |  |
| AbAi-2×GCC-F | GGTACCAGCCGCCAGCCGCCAGCCGCCAGCCGCCAGCCGCC | Yeast-one-hybrid |
| AbAi-2×GCC-R | CCTCGAGGGCGGCTGGCGGCTGGCGGCTGGCGGCTGGCGGCT |  |
